# DamMet: ancient methylome mapping accounting for errors, true variants, and post-mortem DNA damage

**DOI:** 10.1093/gigascience/giz025

**Published:** 2019-04-20

**Authors:** Kristian Hanghøj, Gabriel Renaud, Anders Albrechtsen, Ludovic Orlando

**Affiliations:** 1 Lundbeck Foundation GeoGenetics Center, University of Copenhagen, Øster Voldgade 5-7, 1350K Copenhagen, Denmark; 2 Laboratoire d’Anthropobiologie Moléculaire et d’Imagerie de Synthèse, CNRS UMR 5288, Université de Toulouse III, Paul Sabatier (UPS), 31000 Toulouse, France; 3 Computational and RNA Biology, Department of Biology, University of Copenhagen, 2200 Copenhagen, Denmark, Øster voldgade 5-7, 1350k

**Keywords:** ancient DNA, high-throughput DNA sequencing, methylome, epigenetics, CpG dinucleotide

## Abstract

**Background:**

Recent computational advances in ancient DNA research have opened access to the detection of ancient DNA methylation footprints at the genome-wide scale. The most commonly used approach infers the methylation state of a given genomic region on the basis of the amount of nucleotide mis-incorporations observed at CpG dinucleotide sites. However, this approach overlooks a number of confounding factors, including the presence of sequencing errors and true variants. The scale and distribution of the inferred methylation measurements are also variable across samples, precluding direct comparisons.

**Findings:**

Here, we present DamMet, an open-source software program retrieving maximum likelihood estimates of regional CpG methylation levels from ancient DNA sequencing data. It builds on a novel statistical model of post-mortem DNA damage for dinucleotides, accounting for sequencing errors, genotypes, and differential post-mortem cytosine deamination rates at both methylated and unmethylated sites. To validate DamMet, we extended gargammel, a sequence simulator for ancient DNA data, by introducing methylation-dependent features of post-mortem DNA decay. This new simulator provides direct validation of DamMet predictions. Additionally, the methylation levels inferred by DamMet were found to be correlated to those inferred by epiPALEOMIX and both on par and directly comparable to those measured from whole-genome bisulphite sequencing experiments of fresh tissues.

**Conclusions:**

DamMet provides genuine estimates for local DNA methylation levels in ancient individual genomes. The returned estimates are directly cross-sample comparable, and the software is available as an open-source C++ program hosted at https://gitlab.com/KHanghoj/DamMet along with a manual and tutorial.

## Introduction

Recent studies in ancient DNA (aDNA) research have demonstrated that osseous methylomes can be mapped from the high-throughput DNA sequencing (HTS) data underlying ancient genomes [1–3]. This paves the way for identifying potentially evolutionary-relevant epigenetic changes during major environmental and societal transitions [4]. Although aDNA methylation states can be inferred following methods usually applied to fresh tissues such as bisulfite DNA sequencing [5] and methyl-binding domains enrichment [6], the degraded nature of aDNA molecules generally limits methylome mapping to indirect computational proxies exploiting post-mortem DNA deamination (PMD) footprints at CpG dinucleotides.

Two available software programs, epiPALEOMIX [1] and ROAM [2], have been recently developed to map DNA methylation levels at the regional scale. Specifically, they leverage the observation that post-mortem cytosine deamination is faster at methylated than unmethylated CpGs [6,7], which leaves an excess of CpG→TpG conversions at methylated sites. Both programs recover statistical measures for regional DNA methylation levels from the counts of CpG→TpG mis-incorporations observed in an ancient genome, relative to a reference genome. The method accuracy can be especially improved when molecular tools are used to eliminate CpG→TpG mis-incorporations introduced following PMD at unmethylated cytosines [1]. Although the available methodologies have been successful in retrieving epigenetic information from ancient individuals, they have a number of drawbacks. They overlook the possible presence of (i) true sequence variants in CpG contexts, (ii) mapping and sequencing errors, (iii) remaining PMD footprints at unmethylated CpGs [8], and (iv) uneven PMD rates along aDNA molecules [8]. Last, the regional methylation scores returned are neither readily cross-sample comparable nor directly comparable to methylation data generated using methods applied to fresh tissues.

Herein, we present DamMet, a software program returning regional maximum likelihood estimates (MLEs) of CpG methylation from HTS data obtained from individual ancient specimens. The underlying algorithm follows a 2-step procedure, the first step of which aims at obtaining MLEs of PMD rates in a position-specific manner at both methylated and unmethylated CpG dinucleotides. To disentangle PMD events at such sites, we assume that the expected fraction of methylated cytosines genome-wide is known. In mammals, the fraction of methylated CpG dinucleotides in somatic tissues is 70−80% [9], implying that 20−30% of unmethylated states can be expected at CpG sites. The second step makes use of the deamination rates obtained in the first step to recover an MLE of *f*, the fraction of methylated cells in any given genomic window (together with a 95% confidence interval).

DamMet tackles all of the above-mentioned limitations of the computational packages currently available for mapping ancient methylomes [1,2]. In particular, DamMet relies on a new, more realistic statistical model of post-mortem DNA deamination at CpG sites, which integrates the actual deamination rates along a DNA fragment (per read group, if needed) for both methylated and unmethylated cytosines (Supplementary Methods 1.2). It accounts for the presence of true variants in an unobserved dinucleotide genotype space and handles both sequencing and mapping errors, all in a probabilistic manner. Finally, MLEs of *f* are directly comparable to those measured from modern methylation data (e.g., whole-genome bisulphite sequencing [WGBS] data) and between ancient samples, leaving no need for further normalization and/or statistical rescaling (Supplementary Methods 1.3).

## Materials and Methods

In this section, we give an overview on the 2-step algorithm implemented in DamMet (for an in-depth description of the entire model, see Supplementary Methods 1.2 and 1.3).

In the first step, we obtain an MLE of PMD rates (*D*) at both methylated and unmethylated cytosines. These rates are position-specific along DNA fragments to account for differential deamination within overhanging ends and the double-stranded parts of aDNA molecules [10]. The full likelihood function leverages chromosome-wide read observations ($\mathbb {D}$) covering cytosines in the reference genome, including equal amounts of those within and outside CpGs:

(1) \begin{eqnarray*}
L(D| \mathbb {D}) = \prod_{j=1}^{J} \prod_{i=1}^{I} p(X_{j,i,k,v} | D_{M,k,v}, Q_{j,i}, \epsilon_{j,i}, F_{\text{global}}) \, ,
\end{eqnarray*}

where *D*_*M,k,v*_ denotes the post-mortem cytosine deamination rate at read position *k* from the 5′ or 3′ (*v*) of a DNA fragment, within methylated (*M* = 1) or unmethylated contexts (*M* = 0). Additionally, *Q_j,i_* is the probability of a mapping error for a given DNA fragment *i* at site *j*, ϵ_*j,i*_ is the probability of a sequencing error at observation *X*_*j,i,k,v*_, and *F*_global_ is the user-defined overall fraction of methylated cytosines, which defaults to 0.75.

The second step makes use of *D*, obtained in the first step, to recover an MLE of *f*, the fraction of methylated CpGs in a given genomic window. The likelihood function of *f* incorporates all sequencing data $(\mathbb {D})$ overlapping a set of genomic CpG dinucleotides (*S*):

(2) \begin{eqnarray*}
L(f | \mathbb{D}) = \prod_{S} \sum_{G \in (0 \ldots 6)} p(G=g) p(X | f,G=g, D,\theta) \,,
\label{eq:full_like_f}
\end{eqnarray*}

where *p*(*X*|*f, G* = *g, D*, θ) is the probability of the dinucleotide pile of sequencing reads (*X*) at a site *s* given *f*, considering an unobserved dinucleotide genotype *g*, and the position-specific deamination rates (*D*). *p*(*G* = *g*) is the prior probability of the unobserved dinucleotide genotype.

The algorithm implemented in DamMet should ideally be tested against simulated data for which the results are known. In the absence of a simulator reproducing the characteristics of aDNA methylation, we have developed a new version of the gargammel simulator [11], which integrates methylation-specific and position-specific PMD patterns. The methodology consists first of simulating sequencing data from an arbitrary number of 100 diploid genomes, where each CpG position is flagged as methylated or unmethylated on the basis of user-provided methylation maps. Post-mortem damage is then added using position-specific deamination matrices at methylated or unmethylated sites, and finally, adapters are added. Cytosine deamination rates outside CpG contexts are assumed to follow those of unmethylated cytosines within CpG contexts. For an in-depth description of the sequence simulator, see Supplementary Results 2.1.1.

## Results

We first tested the accuracy of both steps of the model implemented in DamMet using simulated data with gargammel and following the methodology described above. Specifically, we simulated sequencing data with 3 different deamination profiles spanning a range of PMD rates: the 4,000-year-old Saqqaq Palaeo-Eskimo [12], the 36,000-year-old Kostenki14 individual [13], and the 45,000-year-old Ust’Ishim specimen [14] (Supplementary Results 2.1). Simulated data are hereafter referred to using the "S-" prefix. The 3 simulated examples allowed us to test the accuracy of DamMet to obtain known deamination profiles from samples generated with different wet-lab procedures, including the most commonly used double-stranded [15] (Saqqaq and Kostenki14) and single-stranded [16] DNA library preparation protocols (Ust’Ishim).

**Figure 1:**
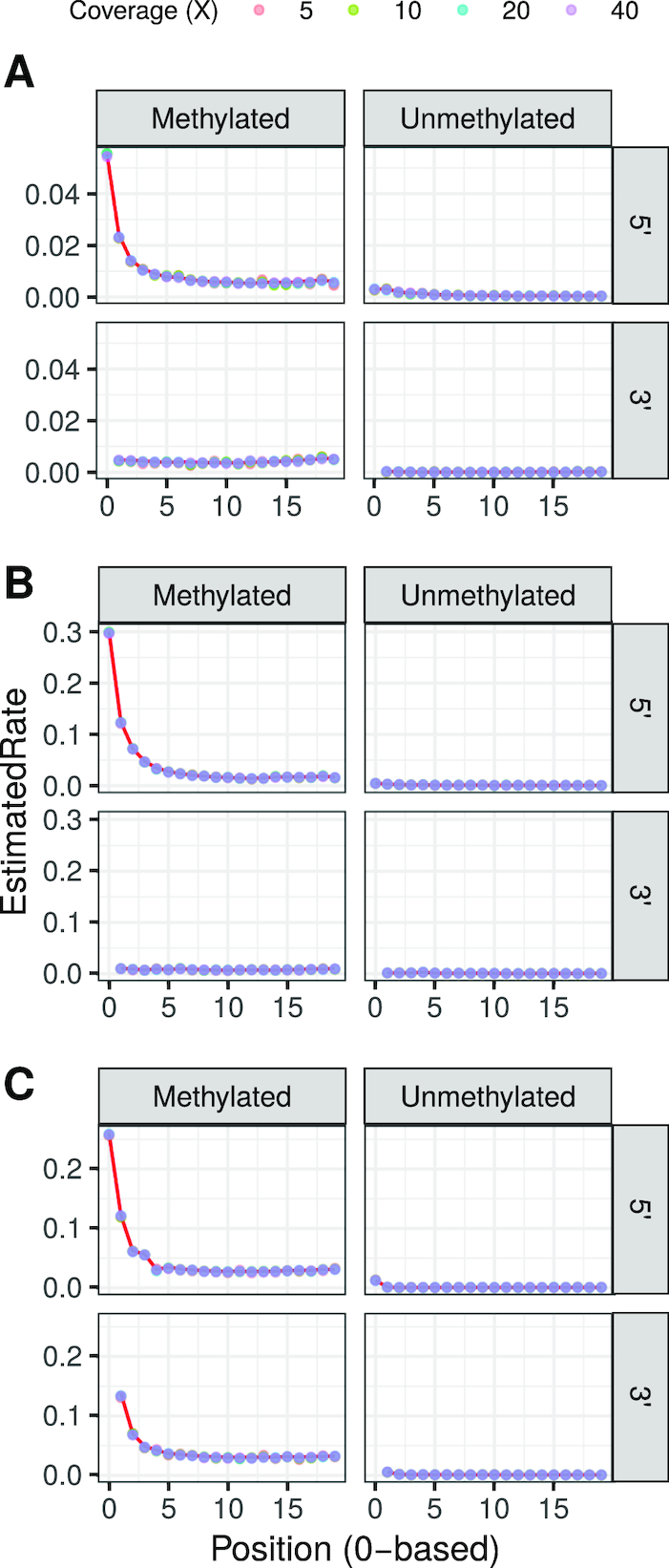
Estimated deamination rates from simulated sequencing data of S-Saqqaq (a), S-Kostenki14 (b), and S-Ust’Ishim (c) at (un)methylated cytosines located in the first 20 positions of the 5′ and 3′ termini of a DNA molecule across a range of possible coverages (X-fold). Known deamination rates are shown as a red line.

More specifically, we applied the first step of the model implemented in DamMet, which is aimed at estimating position-specific PMD rates at both methylated and unmethylated sites, to the 3 simulated datasets across a wide range of genome coverage. To obtain the MLE of methylation position-specific PMD rates, the likelihood function makes use of chromosome-wide read observations covering a cytosine located in a CpG context in the reference genome and an equal number of observations of cytosines located outside a CpG context. We find that the MLEs of position-specific PMD rates at both methylated and unmethylated cytosines (*D*) are highly accurate, at least down to 5-fold coverage, regardless of the PMD profile and library protocol considered (Fig. 1 and Supplementary Results Section 2.1.3). These results validate the first step of the algorithm.

**Figure 2:**
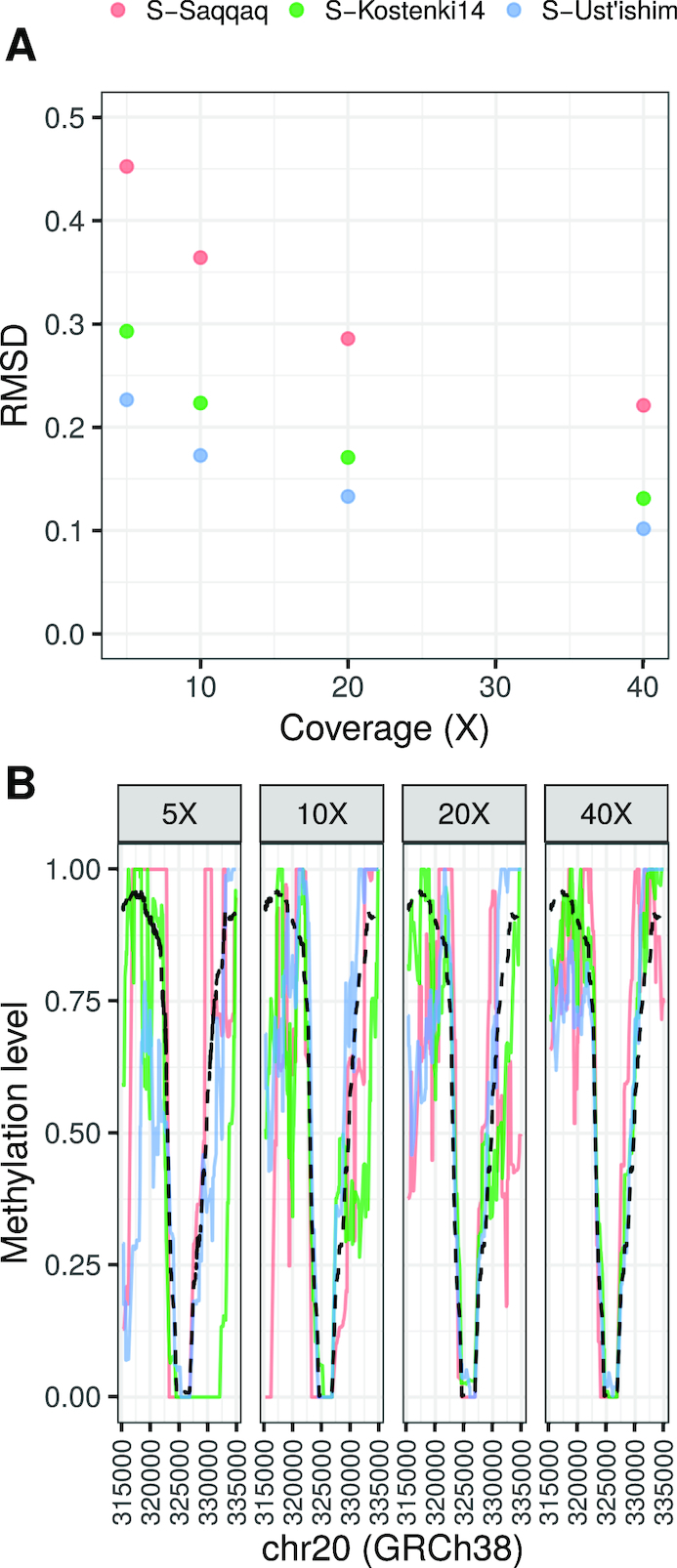
**(a)** Root mean square deviation (RMSD) of *f* estimates (estimated_*f* – known_methylation) for a coverage range (5–40×) and a window size of 50 CpGs for the 3 simulated datasets. **(b)** Estimates of *f* in a local genomic region with a window size of 50 CpGs and a coverage range (5–40×). The expected methylation profile is shown as a black dashed line.

Next, we analyzed the accuracy of the second step, estimating local methylation levels (*f*), across a wide range of genomic window sizes and sequencing efforts using the same 3 simulated datasets (Supplementary Results 2.1). Here, the likelihood function is maximized to obtain *f*, and includes all dinucleotide read observations covering CpGs in a given genomic window. First, we investigated the accuracy of *f* across a range of sequencing efforts using the root mean squared deviation of the estimates as measures of accuracy (Fig. 2a) (see Supplementary Results 2.1.4 for a wide range of combinations of diverse sequencing efforts and window sizes). We found that the DamMet accuracy increases with sequencing depth (and/or window size) in all 3 scenarios. Additionally, we found that the accuracy is positively correlated with PMD levels. Both observations are in line with the expectations of our likelihood model. We also compared *f* with the true methylation levels within a CpG island (chr20:324243–327679; GRCh38) for the same simulated datasets and sequencing depths. This specific CpG island was selected to illustrate the abrupt decline in methylation levels often observed in CpG islands. We found that the accuracy of the MLE of *f* increased with higher sequencing depths (Fig. 2B) and that in all simulated scenarios, confident estimates of *f* require ≥20× coverage. Further information about the trade-off between accuracy of *f* and resolution by permuting a range of window sizes and sequencing efforts both locally and chromosome-wide can be found in Supplementary Results 2.1.4. Finally, we demonstrated that DamMet obtains accurate methylation estimates in regions with a high density of true variants located in CpG contexts by incorporating the possibility of observing true dinucleotide variants in the likelihood function (Supplementary Results 2.1.5).

**Figure 3:**
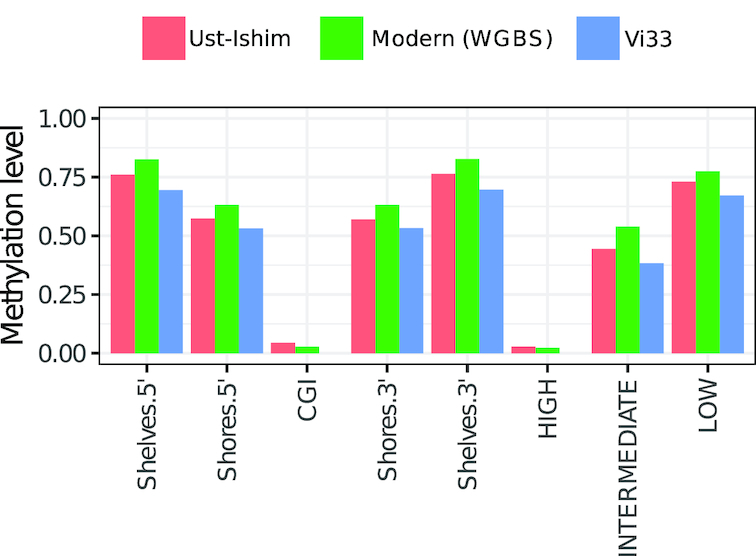
Genomic regions with contrasted methylation levels including CpG islands (CGI), their shores and shelves, and promoter regions stratified by their percent guanine and cytosine content and CpG density (high, intermediate, and low). Modern (green) is provided as a comparative baseline using methylation data retrieved from a fresh somatic adipose tissue sample.

Finally, we further validated DamMet using the sequencing data from 2 ancient specimens: the 45,000-year-old Ust’Ishim (42-fold coverage; 17) [14] and the 50,000-year-old Vi33 Neanderthal (30-fold coverage; 18) [19] (Supplementary Results 2.2). All DNA libraries for Ust’Ishim were prepared on uracil-specific excision reagent (USER)-treated DNA extracts (NEB, USA). Following this enzymatic treatment, almost all PMD events (C→T) derive from methylated cytosines [8]. In contrast, the Vi33 sample consists of only a single DNA library prepared following USER treatment and 8 DNA libraries prepared in absence of treatment (Supplementary Results 2.2.2). In the latter libraries, both methylated and unmethylated cytosines are sequenced as thymines, which confounds the methylation estimate if not properly accounted for. By analyzing Vi33, we can thus test the ability of DamMet to obtain reliable estimates of *f* despite the presence of similar conversion signals at both methylated and unmethylated cytosine residues. We found that our chromosome-wide MLEs of *f* are comparable (Ust’Ishim: 0.768, Vi33: 0.756) to the methylation levels measured in modern samples generated with WGBS (Modern: 0.748). The same holds true at the regional level, where DamMet obtains methylation estimates highly similar to those found in WGBS data from a modern sample (Fig. 3). Both samples display a minor underestimate (RMSD: 0.04-0.05) due to the relatively small genomic window sizes. Importantly, we also demonstrate that reliable methylation estimates can be obtained from non-USER–treated specimen data.

Finally, we investigated whether DamMet retrieved regional methylation values on par with the Ms count statistic implemented in epiPALEOMIX [1]. Because Ms is not scaled and thus not directly comparable in terms of absolute values, we instead investigated the correlation of Ms and *f* in various genomic regions (Table 1). We find that for both Ust-Ishim and Vi33, the 2 statistics are positively correlated (p-values <1*e*^−16^). In line with our expectation, we find much lower positive correlation coefficients for Vi33 than for Ust-Ishim because epiPALEOMIX assumes that all observed CpG→TpG conversions, including sequencing errors, true variants, and deamination of unmethylated cytosine residues, reflect true signals of methylation.

**Table 1:** Pearson correlation of MLE of *f* and Ms in genomic regions

Genomic region	Ust-Ishim	Vi33
Shelves.5′	0.4935	0.2729
Shores.5′	0.5704	0.3278
CGI	0.8345	0.4403
Shores.3′	0.6974	0.3306
Shelves.3′	0.5315	0.2686
High	0.5944	0.3415
Intermediate	0.8334	0.4263
Low	0.4347	0.2501

Note: all P-values <1*e*^−16^.

## Conclusion

DamMet provides a new statistical method to obtain reliable estimates of methylation levels that are directly comparable between ancient and modern samples. It is robust to the presence of true genotype variants, takes mapping and sequencing errors into account, and facilitates analyses of non-USER–treated sequencing data by estimating the position-specific deamination rates at both methylated and unmethylated CpG dinucleotides.

By combining DamMet and the novel sequencing simulator, a qualified estimate of the necessary sequencing efforts and/or window sizes to recover reliable *f* estimates can be obtained accounting for the specific properties of any given ancient sample (e.g., PMD levels and/or read length distribution). Thus, the optimal trade-off between the accuracy of *f* and resolution in terms of genomic window size can be quantified.

Given that the vast majority of high-coverage ancient genomes are from human and domesticated animal specimens, the current implementation of DamMet estimates methylation in symmetric CpG contexts. This is by far the most dominant context associated with methylation in mammals. Nonetheless, following an increase in high-coverage ancient genomes for which methylation occurs predominantly in asymmetric sequence contexts (e.g., CpNpN, which often occurs in plants [20]), MLE estimates of *f* in such contexts could be added in future releases of DamMet.

## Implementation Details

DamMet software is implemented in C++ and can be found at [21] https://gitlab.com/KHanghoj/DamMet. It takes a BAM file [22] as input, together with the reference genome used for read alignment. Three canonical filters for HTS data, namely, base quality, mapping quality, and minimum DNA fragment length, are implemented in DamMet [23]. These can be modified by the user. Moreover, DamMet ignores "N" nucleotides present in either the reference genome or sequencing data.

The reconstruction of the entire methylome of Ust-Ishim and Vi33 took 11 and 8 hours on a single CPU (E5-2683 v4 at 2.10 GHz) with memory usages peaking at 10 and 9GB, respectively. Because DamMet analyzes each chromosome individually, it can easily be parallelized per chromosome to speed up the computation time. Regional methylation levels can be recovered using either a sliding window procedure along the chromosome or within genomic regions based on a user-provided BED file. Low-mappability regions can be masked prior to estimating the regional methylation level by providing the regions in a BED format file. Particular genomic sites can also be excluded if needed. Along with DamMet, 2 dependencies will be installed, namely, nlopt and htslib.

The new sequence simulator, implemented as a novel feature in gargammel [11], is available at https://github.com/grenaud/gargammel, together with a manual and running examples.

## Availability of source code and requirements

- Project name: DamMet
- Project home page: https://gitlab.com/KHanghoj/DamMet
- Operating system(s): platform indepedent
- Programming language: C++
- Other requirements: htslib, nlopt
- License: MIT
- RRID: SCR_016959

## Availability of supporting data and materials

Supporting data and an archival copy of the code are available via the GigaScience repository GigaDB [24].

## Additional file

**Supplementary information**: Supplementary Methods and Results are available via the additional file associated with this article.

GIGA-D-18-00422_Original_Submission.pdf

GIGA-D-18-00422_Revision_1.pdf

GIGA-D-18-00422_Revision_2.pdf

Response_to_Reviewer_Comments_Original_Submission.pdf

Response_to_Reviewer_Comments_Revision_1.pdf

Reviewer_1_Report_Original_Submission -- Alexander Peltzer 12/2/2018 Reviewed

Reviewer_1_Report_Revision_1 -- Alexander Peltzer 2/12/2019 Reviewed

Reviewer_2_Report_Original_Submission -- Diogo Pratas 1/25/2019 Reviewed

Reviewer_2_Report_Revision_1 -- Diogo Pratas 2/13/2019 Reviewed

Supplemental File

## Abbreviations

aDNA: ancient DNA; HTS: high-throughput DNA sequencing; MLE: maximum likelihood estimate; PMD: post-mortem DNA deamination; RMSD: root mean square deviation; USER: uracil-specific excision reagent; WGBS: whole-genome bisulphite sequencing.

## Competing interests

The authors declare that they have no competing interests.

## Funding

This work was supported by the Danish National Research Foundation (Grant DNRF94), the Villum Fonden miGENEPI research project, and the Initiative d’Excellence Chaires d’attractivité, Université de Toulouse (OURASI). This project has received funding from the European Research Council (ERC) under the European Union’s Horizon 2020 research and innovation programme (grant agreement No. 681605).

## Author’s contributions

K.H. developed the model with input from G.R., A.A., and L.O. K.H. implemented the model and ran all analyses. G.R. implemented the novel sequence simulator. K.H. and L.O. wrote the manuscript with input from all authors.
