# Supplementary material for: DamMet: ancient methylome mapping accounting for errors, true variants, and post-mortem DNA damage
Source: Gigascience. 2019 Apr 20;8(4):giz025. doi: 10.1093/gigascience/giz025 (PMC6474913; doi:10.1093/gigascience/giz025)
Supplement: GIGA-D-18-00422_Original_Submission.pdf [file giz025_giga-d-18-00422_original_submission.pdf]

GigaScience

DamMet, a full probabilistic model for mapping ancient methylomes

--Manuscript Draft--

**Manuscript Number:**	GIGA-D-18-00422
**Full Title:**	DamMet, a full probabilistic model for mapping ancient methylomes
**Article Type:**	Technical Note
**Funding Information:**	H2020 European Research Council () (681605) — PhD Ludovic Orlando<br>Strategiske Forskningsråd (DNRF94) — PhD Ludovic Orlando<br>OURASI (OURASI) — PhD Ludovic Orlando
**Abstract:**	Background: Recent computational advances in ancient DNA research have opened access to the detection of ancient DNA methylation footprints at genome-wide scales. The most common approach infers the methylation state of CpG dinucleotides based on their patterns of post-mortem DNA damage. However, it overlooks a number of factors impacting the methylome reconstruction, such as sequencing errors and true variants. The scale and distribution of the inferred methylation levels are also variable across samples, precluding direct comparisons.<br>Results: Here, we present DamMet, an open-source software retrieving maximum likelihood estimates of regional CpG methylation levels from ancient DNA sequencing data. It builds on a novel statistical model of post-mortem DNA damage for dinucleotides, accounting for sequencing errors, dinucleotide genotypes, and differential post-mortem deamination rates at methylated and unmethylated sites. In order to validate DamMet, we extended gargammel, a sequence simulator for ancient DNA data, by incorporating methylation-dependent features of post-mortem DNA decay. Besides being validated using the novel simulator, DamMet also obtains methylation levels on par and directly comparable to that generated with whole genome bisulphite sequencing from fresh tissues.<br>Conclusions: DamMet, a software to obtain cross-sample comparable methylation data from ancient specimen, is available as an open source C++ program hosted at https://gitlab.com/KHanghoj/DamMet along with a manual and tutorial.
**Corresponding Author:**	Kristian Hanghøj<br><br>DENMARK
**Corresponding Author Secondary Information:**	
**Corresponding Author's Institution:**	
**Corresponding Author's Secondary Institution:**	
**First Author:**	Kristian Hanghøj
**First Author Secondary Information:**	
**Order of Authors:**	Kristian Hanghøj
	Gabriel Renaud
	Anders Albrechtsen
	Ludovic Orlando

Powered by Editorial Manager® and ProduXion Manager® from Aries Systems Corporation

Order of Authors Secondary Information:	
Additional Information:	
Question	Response
Are you submitting this manuscript to a special series or article collection?	No
**Experimental design and statistics**<br><br>Full details of the experimental design and statistical methods used should be given in the Methods section, as detailed in our Minimum Standards Reporting Checklist. Information essential to interpreting the data presented should be made available in the figure legends.<br><br>Have you included all the information requested in your manuscript?	Yes
**Resources**<br><br>A description of all resources used, including antibodies, cell lines, animals and software tools, with enough information to allow them to be uniquely identified, should be included in the Methods section. Authors are strongly encouraged to cite Research Resource Identifiers (RRIDs) for antibodies, model organisms and tools, where possible.<br><br>Have you included the information requested as detailed in our Minimum Standards Reporting Checklist?	Yes
**Availability of data and materials**<br><br>All datasets and code on which the conclusions of the paper rely must be either included in your submission or deposited in publicly available repositories (where available and ethically appropriate), referencing such data using a unique identifier in the references and in the "Availability of Data and Materials" section of your manuscript.	Yes

Powered by Editorial Manager® and ProduXion Manager® from Aries Systems Corporation

Have you have met the above requirement as detailed in our Minimum Standards Reporting Checklist?	

Manuscript
Click here to download Manuscript main.tex

```
This is pdfTeX, Version 3.14159265-2.6-1.40.19 (TeX Live 2018/W32TeX)
(preloaded format=pdflatex 2018.7.12)  29 OCT 2018 11:28
entering extended mode
 restricted \write18 enabled.
 %&-line parsing enabled.
**main.tex
(./main.tex
LaTeX2e <2018-04-01> patch level 5

! LaTeX Error: File `oup-contemporary.cls' not found.

Type X to quit or <RETURN> to proceed,
or enter new name. (Default extension: cls)

Enter file name:
! Emergency stop.
<read *>

l.11 ^^M

*** (cannot \read from terminal in nonstop modes)


Here is how much of TeX's memory you used:
 10 strings out of 492646
 215 string characters out of 6133325
 56709 words of memory out of 5000000
 3994 multiletter control sequences out of 15000+600000
 3640 words of font info for 14 fonts, out of 8000000 for 9000
 1141 hyphenation exceptions out of 8191
 10i,0n,8p,86b,8s stack positions out of 5000i,500n,10000p,200000b,80000s
!  ==> Fatal error occurred, no output PDF file produced!
```

Manuscript
Click here to download Manuscript dammet-full-probabilistic.pdf

OXFORD (GIGA)n SCIENCE

GigaScience, 2018, 1–5

doi: xx.xxxx/xxxx
Manuscript in Preparation
Technical Note

TECHNICAL NOTE

DamMet, a full probabilistic model for mapping ancient methylomes

Kristian Hanghøj[1,2,*], Gabriel Renaud[1,2], Anders Albrechtsen[3] and Ludovic Orlando[1.2,*]

[1]Centre for GeoGenetics, Natural History Museum of Denmark, University of Copenhagen, 1350K Copenhagen, Denmark and [2]Laboratoire d'Anthropologie Moléculaire et d'Imagerie de Synthèse, CNRS UMR 5288, Université de Toulouse, University Paul Sabatier (UPS), 31000 Toulouse, France and [3]Computational and RNA Biology, Department of Biology, University of Copenhagen, 2200 Copenhagen, Denmark

[*] Corresponding authors kristianhanghoej@gmail.com and ludovic.orlando@univ-tlse3.fr

Abstract

Background: Recent computational advances in ancient DNA research have opened access to the detection of ancient DNA methylation footprints at genome-wide scales. The most common approach infers the methylation state of CpG dinucleotides based on their patterns of post-mortem DNA damage. However, it overlooks a number of factors impacting the methylome reconstruction, such as sequencing errors and true variants. The scale and distribution of the inferred methylation levels are also variable across samples, precluding direct comparisons.
Results: Here, we present DamMet, an open-source software retrieving maximum likelihood estimates of regional CpG methylation levels from ancient DNA sequencing data. It builds on a novel statistical model of post-mortem DNA damage for dinucleotides, accounting for sequencing errors, dinucleotide genotypes, and differential post-mortem deamination rates at methylated and unmethylated sites. In order to validate DamMet, we extended gargammel, a sequence simulator for ancient DNA data, by incorporating methylation-dependent features of post-mortem DNA decay. Besides being validated using the novel simulator, DamMet also obtains methylation levels on par and directly comparable to that generated with whole genome bisulphite sequencing from fresh tissues.
Conclusions: DamMet, a software to obtain cross-sample comparable methylation data from ancient specimen, is available as an open source C++ program hosted at https://gitlab.com/KHanghoj/DamMet along with a manual and tutorial.
Supplementary information: Supplementary Methods and Results are available at XXX.

Key words: Ancient DNA; High-Throughput DNA sequencing; methylome; epigenetics

Introduction

Recent studies in ancient DNA (aDNA) research have demonstrated that methylomes can be recovered from ancient specimens [1, 2, 3]. This paves the way for monitoring evolutionary-relevant epigenetic changes during major environmental and societal transitions [4]. Although aDNA methylation data can be retrieved following methods usually applied to fresh tissues, the degraded nature of aDNA molecules generally limits methylome mapping to indirect computational proxies exploiting post-mortem DNA deamination (PMD) footprints at CpG dinucleotides.

The available software epiPALEOMIX [1] and ROAM [2] exploit the counts of CpG→TpG mis-incorporations observed in an ancient genome, relative to a reference genome, as signatures of CpG methylation. Post-mortem cytosine deamination is faster at methylated than unmethylated CpGs [5, 6], leaving an excess of CpG→TpG conversions when methylation is present. This difference can be magnified, and the method' accuracy improved, when molecular tools are used to remove the

Compiled on: October 29, 2018.
Draft manuscript prepared by the author.

deamination by-product of unmethylated cytosines [1]. However, the available methods have a number of caveats. They overlook the possible presence of (1) true sequence variants in CpG contexts, (2) mapping and sequencing errors, (3) remaining PMD footprints at unmethylated CpGs [7], and (4) uneven PMD rates along aDNA molecules [7]. Lastly, the regional methylation score is neither readily cross-sample comparable nor directly comparable to methylation data generated using methods applied to fresh tissues.

Here, we present DamMet, a software returning regional maximum likelihood estimates (MLE) of CpG methylation from high-throughput DNA sequencing data obtained from an ancient specimen. The algorithm follows a two step procedure, where the first step aims at obtaining MLE of the PMD rates in a position-specific manner at both methylated and unmethylated cytosines. To disentangle deamination events of a methylated cytosines from unmethylated cytosines, we use a prior reflecting the expected fraction of methylated cytosines genome-wide. In mammals, the fraction of methylated cytosines in CpG context in somatic tissues is 70 – 80% [8], meaning that 20 – 30% are expected to be found in unmethylated states. The second step makes use of the deaminatino rates obtained in the first step to recover a MLE of f, the fraction of methylated cells in any given genomic window (together with a 95% confidence interval).

DamMet tackles all of the above-mentioned limitations of the computational packages currently available for mapping ancient methylomes [2, 1]. In particular, DamMet relies on a new, more realistic statistical model of post-mortem DNA deamination at CpG sites, which integrates the actual deamination rates along a DNA fragment (per read group, if needed) both for methylated and unmethylated cytosines (Supplementary Methods 1.2), consider true variants in an unobserved dinucleotide genotype space, and handles sequencing and mapping errors all in a probabilistic manner. Lastly, MLEs of f are directly comparable to these measured from modern methylation data (e.g. WGBS data) and between ancient samples, leaving no need for further normalization and/or statistical scaling (Supplementary Methods 1.3).

Materials and Methods

In this section, we give an intuition on the two step algorithm implemented in DamMet (for an indepth description of the entire model see Supplementary Methods 1.2 and 1.3).

In the first step, we obtain a MLE of PMD rates (D) at both methylated and unmethylated cytosines. These rates are position-specific along DNA fragments to account for differential deamination within overhanging and double-stranded parts of aDNA molecules [9]. The full likelihood function leverages chromosome-wide read observations ($\mathbb{D}$) covering cytosines in the reference genome, including equal amounts of those within and outside CpGs:

$$L(D|\mathbb{D}) = \prod_{j=1}^{sites} \prod_{i=1}^{depth} p(X_{j,i,k,v}|D_{M,k,v}, Q_{j,i}, \epsilon_{j,i}, F_{global}) \quad (1)$$

where $D_{M,k,v}$ denotes the post-mortem cytosine deamination rate at read position k from the 5' or 3'prime (v) of a DNA fragment, within a methylated ($M = 1$) or an unmethylated context ($M = 0$). Additionally, $Q_{j,i}$ is the probability of a mapping error for DNA fragment (i) at site j, $\epsilon_{j,i}$ is the probability of a sequencing error at observation $X_{j,i,k,v}$, and F_{global} is the user defined overall fraction of methylated cytosines, default to 0.75.

The second step makes use of D, obtained in the first step, to recover a MLE of f, the fraction of methylated CpGs in a given genomic window. The likelihood function of f incorporates all sequencing data ($\mathbb{D}$) overlapping a set of genomic CpG dinucleotides (S):

$$L(f|\mathbb{D}) = \prod_{S} \sum_{G \in (0...6)} p(G = g)p(X|f, G = g, D, \theta) \quad (2)$$

where $p(X|f, G = g, D, \theta)$ is the probability of the dinucleotide pile of sequencing reads (X) at a site s given f, considering an unobserved dinucleotide genotype g, and the position-specific deamination rates (D). $p(G = g)$ is the prior probability of the unobserved dinucleotide genotype.

The algorithm implemented in DamMet should ideally be tested against simulated data where the results are known. In absence of a simulator reproducing the characteristics of aDNA methylation, we have developed a new version of the gargammel simulator [10] integrating methylation-specific and position-specific PMD patterns. The methodology consists first of simulating sequencing data from 100 diploid genomes, where each CpG position is flagged as methylated or unmethylated. Post-mortem damage is then added using position-specific deamination matrices at methylated or unmethylated sites and lastly, adapters are added. Cytosine deamination rates outside CpG contexts are assumed to follow those of unmethylated cytosines within CpG contexts. For an in-depth description of the sequence simulator see Supplementary Results 2.1.1.

Results

We first tested the accuracy of both steps of the model implemented in DamMet using simulated data following the methodology described above. Specifically, we simulated sequencing data (prefix: 'S-') with three different deamination profiles spanning a range of PMD rates: the 4k year-old Saqqaq Palaeo-Eskimo [11], the 36k year-old Kostenki14 individual [12], and the 45k year-old Ust'ishim specimen [13] (Supplementary Results 2.1). These three simulated examples also allowed us to test the accuracy of DamMet to obtain deamination profiles from samples generated with double stranded [14] (Saqqaq and Kostenki14) and single stranded [15] DNA library preparation protocols (Ust'Ishim).

We applied the first step of the model implemented in DamMet, estimating position-specific PMD rates at both methylated and unmethylated sites, to the three simulated dataset across a wide range of genome coverage. To obtain the MLE of methylation position-specific PMD rates the likelihood function uses chromosome-wide read observations covering a cytosine where a CpG is located in the reference genome and equally many observations of cytosines outside CpG context. We find that the MLE of position-specific PMD rates at both methylated and unmethylated cytosines ($\mathbb{D}$) are highly accurate, at least down to 5-fold coverage, regardless of the deamination profile and library protocol considered (Fig 1 and Supplementary Results Section 2.1.3). These results validate the first step of the algorithm.

Next, we analyzed the accuracy of the second step, estimating local methylation levels (f), across a wide range of genomic window sizes and sequencing efforts using the same three simulated datasets (Supplementary Results 2.1). The likelihood function is maximized to obtain f, includes all dinucleotide read observations covering CpGs in a given genomic window. First, we investigated the accuracy of f across a range of sequencing efforts using the root mean squared deviation of the estimates as measures of accuracy (Fig 2A) (see Supplementary

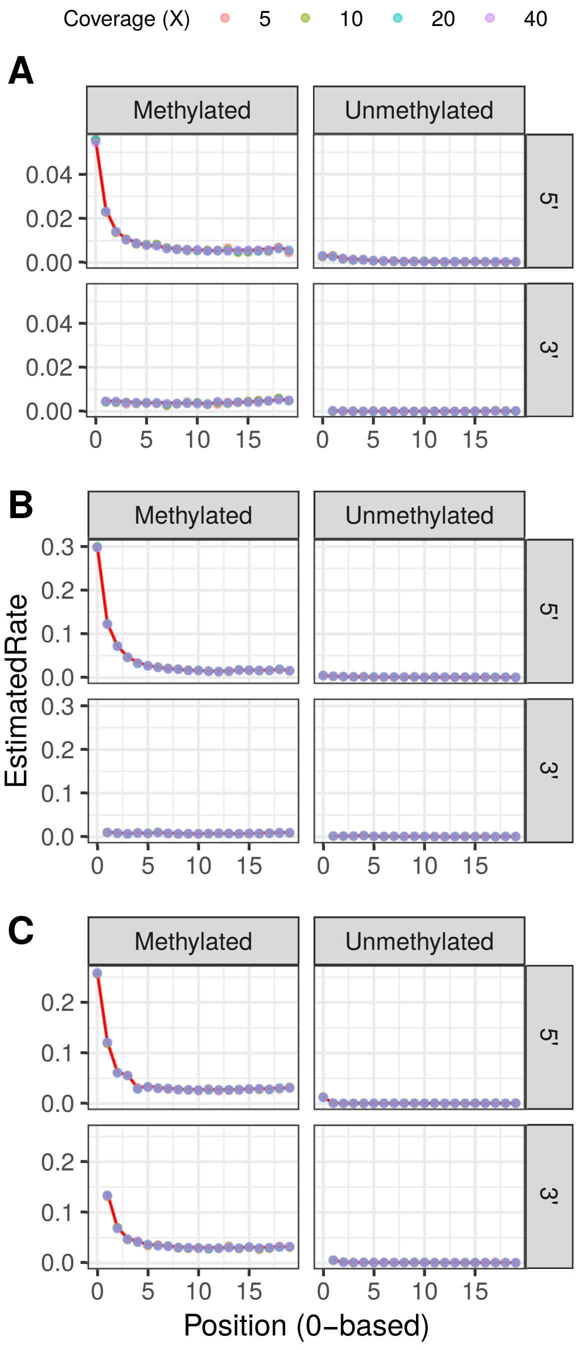

Figure 1. Estimated deamination rates from simulated sequencing data of S-Saqqaq (**A**), S-Kostenki14 (**B**), and S-Ust'ishim (**C**) at (un)methylated cytosines located in the first 20 positions of the 5' and 3' of termini a DNA molecule across a range of possible coverages (X-fold). Known deamination rates are shown as a red line.

Results 2.1.4 for permutations of both sequencing efforts and window sizes). We found that the accuracy increases with sequencing depth (and/or window size) in all three scenarios. Additionally, we found that the accuracy is positively correlated with the PMD levels. Both observations are in line with the expectations of our likelihood model. We also compared f to the true methylation levels within a CpG island (chr20:324243-327679; GRCh38) for the same simulated data sets and sequencing depths. We found the accuracy of the MLE of f increased with higher sequencing depths (Fig 2B). However, in these simulated scenarios, confident estimates of f require at least 20X coverage. Further information about the trade-off between accuracy of f and resolution by permuting a range of window sizes and sequencing efforts both locally and chromosome-wide can be found in the Supplementary Results 2.1.4). Lastly, we demonstrated that DamMet obtains accurate methylation estimates in regions with a high density of true variants located in CpG contexts by incorporating the possibility of observing true dinucleotide variants in the likelihood function (Supplementary Results 2.1.5).

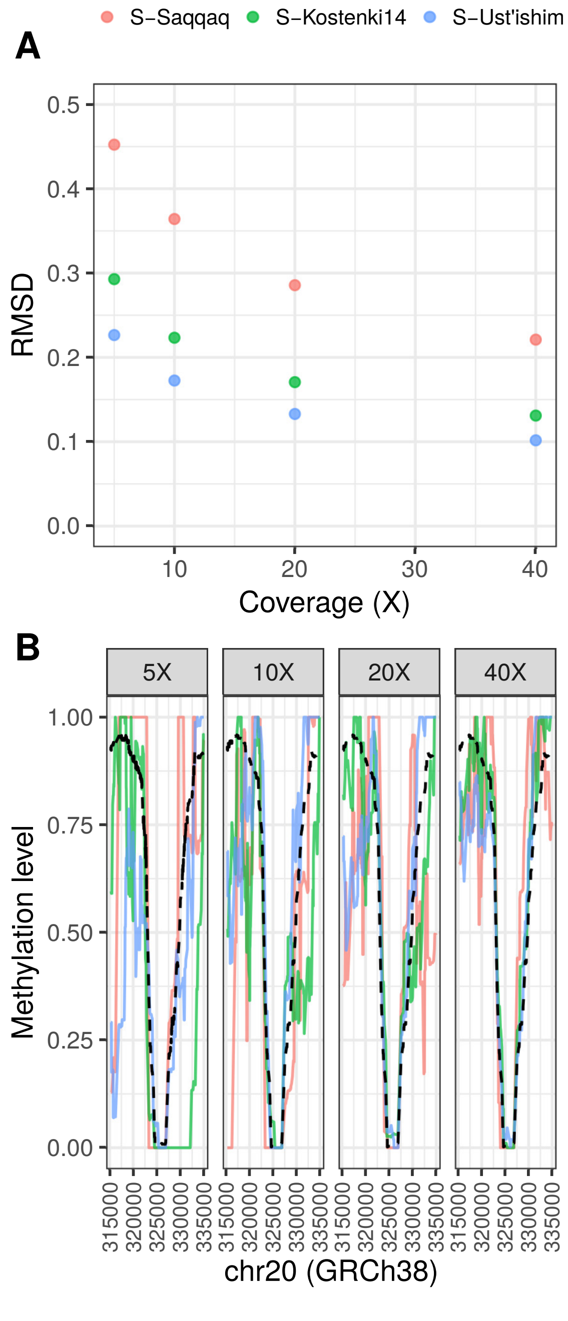

Figure 2. **A** Root mean square deviation (RMSD) of f estimates (Estimated_f - Known_methylation) for a coverage range (5-40X) and a window size of 50 CpGs for the three simulated datasets. **B** Estimates of f in a local genomic with a window size of 50 CpGs and a coverage range (5-40X). The expected methylation profile is shown as a black dashed line

Finally, we tested DamMet using the sequencing data from

two ancient specimens: the 45k year-old Ust'Ishim (42-fold coverage) [13] and the 50k year-old Vi33 Neanderthal (30-fold coverage) [16] (Supplementary Results 2.2). All libraries for Ust'Ishim were prepared on USER-treated DNA extracts. Following this enzymatic treatment, almost all PMD events (C→T) derive from methylated cytosines [7]. In contrast, the Vi33 sample consists of only one single USER treated and eight untreated libraries (Supplementary Results 2.2.2). In the latter libraries, uracil residues (PMD conversion of unmethylated cytosine residues) are also sequenced as thymines, which construes the methylation estimate if it is not corrected for. By analyzing Vi33, we can thus test DamMet ability to obtain reliable estimates of f despite the presence of similar conversion signals at both methylated and unmethylated cytosine residues. We found that our chromosome-wide MLE of f are comparable (Ust'ishim:0.768, Vi33:0.756) to the methylation levels measured in modern samples generated with WGBS (Modern: 0.748). The same holds true at the regional level, where DamMet obtains methylation estimates highly similar to that found in WGBS data from a modern sample (Fig. 3). Both samples display a minor underestimate (RMSD: 0.04-0.05) due to the relatively small genomic window sizes. Importantly, we also demonstrate that reliable methylation estimates can be obtained from non-USER treated data.

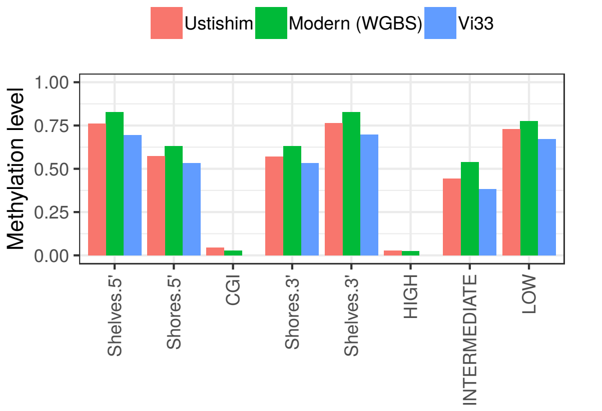

Figure 3. Genomic regions with contrasted methylation levels including CpG islands (CGI), their shores and shelves, and promoter regions stratified by their %GC content and CpG density (HIGH, INTERMEDIATE, and LOW). Modern (green) is provided as a comparative baseline using methylation data retrieved from fresh somatic adipose tissue.

Conclusion

DamMet provides a new statistical method to obtain reliable estimates of methylation levels that are directly comparable between ancient and to modern samples. It is robust to the presence of true variants, accounts of mapping and sequencing errors, and facilitates analyses of non-USER treated sequencing data by estimating the position specific deamination rates at both methylated and unmethylated cytosines.

By combining DamMet and the novel sequencing simulator, a qualified estimate of the necessary sequencing efforts and/or window sizes to recover reliable f estimates can be obtained accounting for the specific properties of any given ancient sample (e.g. PMD levels and/or read length distribution). Thus, the optimal trade off between the accuracy of f and resolution in terms of genomic window size can be quantified.

Implementation Details

DamMet software is implemented in C++ and can be found at https://gitlab.com/KHanghoj/DamMet. It takes a BAM file [17] as input together with the reference genome used for mapping the sample. As DamMet analyze each chromosome individually, it can easily be parallelized per chromosome. For an ancient specimen, sequenced to 30-fold depth-of-coverage, the methylome of human chromosome 1 (GRCh38) was generated in two hours using a single CPU. Regional methylation levels can be recovered either using a sliding window procedure along the chromosome or within genomic regions based on a user-provided BED file. Low mappability regions can be masked prior to estimating the regional methylation level by providing the regions in a BED format file. Along with DamMet, two dependencies will be installed, namely nlopt and htslib.

The new sequence simulator, implemented as a novel feature in gargammel [10], is available at https://github.com/grenaud/gargammel accompanied by a manual and running examples.

Availability of source code and requirements (optional, if code is present)

- Project name: DamMet
- Project home page: https://gitlab.com/KHanghoj/DamMet
- Operating system(s): platform indepedent
- Programming language: c++
- Other requirements: htslib, nlopt
- License: MIT

Availability of supporting materials

Supplementary Methods and Results are available at XXX.

Declarations

List of abbreviations

aDNA: ancient DNA HTS: High throughut sequencing; MLE: maximum likelihood estimate; PMD:post-mortem DNA deamination; WGBS: whole genome bisulphite sequencing data

Competing Interests

None declared

Funding

This work was supported by the Danish National Research Foundation (Grant DNRF94), the Villum Fonden miGENEPI research project, and the Initiative d'Excellence Chaires d'attractivité, Université de Toulouse (OURASI). This project has received funding from the European Research Council (ERC) under the European Union's Horizon 2020 research and innovation programme (grant agreement No 681605). GR was supported by a Marie-Curie Individual Fellowship (MSCA-EF-752657).

Author's Contributions

KH developed the model with input from GR, AA, and LO. KH implemented the model and ran all analyses. GR implemented

the novel sequence simulator. KH and LO wrote the manuscript with input from all authors.

Acknowledgements

We thank the AGES Group members for fruitful discussions.

References

1. Hanghøj K, Seguin-Orlando A, Schubert M, Madsen T, Pedersen JS, Willerslev E, et al. Fast, accurate and automatic ancient nucleosome and methylation maps with epiPALEOMIX. Molecular biology and evolution 2016;33(12):3284–3298.
2. Gokhman D, Lavi E, Prüfer K, Fraga MF, Riancho JA, Kelso J, et al. Reconstructing the DNA methylation maps of the Neandertal and the Denisovan. Science 2014;344(6183):523–527. http://dx.doi.org/10.1126/science.1250368.
3. Pedersen JS, Valen E, Velazquez AMV, Parker BJ, Rasmussen M, Lindgreen S, et al. Genome-wide nucleosome map and cytosine methylation levels of an ancient human genome. Genome research 2014 Mar;24(3):454–466. http://dx.doi.org/10.1101/gr.163592.113.
4. Hanghøj K, Orlando L. Ancient Epigenomics. In: SpringerLink Population Genomics, Springer, Cham; 2018.p. 1–37.
5. Seguin-Orlando A, Hoover CA, Vasiliev SK, Ovodov ND, Shapiro B, Cooper A, et al. Amplification of TruSeq ancient DNA libraries with AccuPrime Pfx: consequences on nucleotide misincorporation and methylation patterns. Science and Technology of Archaeological Research 2015;.
6. Smith RWA, Monroe C, Bolnick DA. Detection of Cytosine methylation in ancient DNA from five native american populations using bisulfite sequencing. PloS one 2015;10(5):e0125344. http://dx.doi.org/10.1371/journal.pone.0125344.
7. Briggs AW, Stenzel U, Meyer M, Krause J, Kircher M, Pääbo S. Removal of deaminated cytosines and detection of in vivo methylation in ancient DNA. Nucleic acids research 2010 Apr;38(6):e87. http://dx.doi.org/10.1093/nar/gkp1163.
8. Li E, Zhang Y. DNA Methylation in Mammals. Cold Spring Harbor Perspectives in Biology 2014 May;6(5). https://www.ncbi.nlm.nih.gov/pmc/articles/PMC3996472/.
9. Briggs AW, Stenzel U, Johnson PLF, Green RE, Kelso J, Prüfer K, et al. Patterns of damage in genomic DNA sequences from a Neandertal. Proceedings of the National Academy of Sciences of the United States of America 2007;104(37):14616–14621. http://dx.doi.org/10.1073/pnas.0704665104.
10. Renaud G, Hanghøj K, Willerslev E, Orlando L. gargammel: a sequence simulator for ancient DNA. Bioinformatics 2017 Feb;33(4):577–579. https://academic.oup.com/bioinformatics/article/33/4/577/2608651.
11. Rasmussen M, Li Y, Lindgreen S, Pedersen JS, Albrechtsen A, Moltke I, et al. Ancient human genome sequence of an extinct Palaeo-Eskimo. Nature 2010;463(7282):757–762. http://dx.doi.org/10.1038/nature08835.
12. Seguin-Orlando A, Korneliussen TS, Sikora M, Malaspinas AS, Manica A, Moltke I, et al. Paleogenomics. Genomic structure in Europeans dating back at least 36,200 years. Science 2014;346(6213):1113–1118. http://dx.doi.org/10.1126/science.aaa0114.
13. Fu Q, Li H, Moorjani P, Jay F, Slepchenko SM, Bondarev AA, et al. Genome sequence of a 45,000-year-old modern human from western Siberia. Nature 2014;514(7523):445–449. http://dx.doi.org/10.1038/nature13810.
14. Meyer M, Kircher M. Illumina sequencing library preparation for highly multiplexed target capture and sequencing. Cold Spring Harbor protocols 2010 Jun;2010(6):db.prot5448. http://dx.doi.org/10.1101/pdb.prot5448.
15. Meyer M, Kircher M, Gansauge MT, Li H, Racimo F, Mallick S, et al. A high-coverage genome sequence from an archaic Denisovan individual. Science 2012;338(6104):222–226. http://dx.doi.org/10.1126/science.1224344.
16. Prüfer K, de Filippo C, Grote S, Mafessoni F, Korlević P, Hajdinjak M, et al. A high-coverage Neandertal genome from Vindija Cave in Croatia. Science 2017;358(6363):655–658.
17. Li H, Handsaker B, Wysoker A, Fennell T, Ruan J, Homer N, et al. The Sequence Alignment/Map format and SAMtools. Bioinformatics 2009;25(16):2078–2079. http://dx.doi.org/10.1093/bioinformatics/btp352.

Figure 1 Click here to download Figure fig1.eps

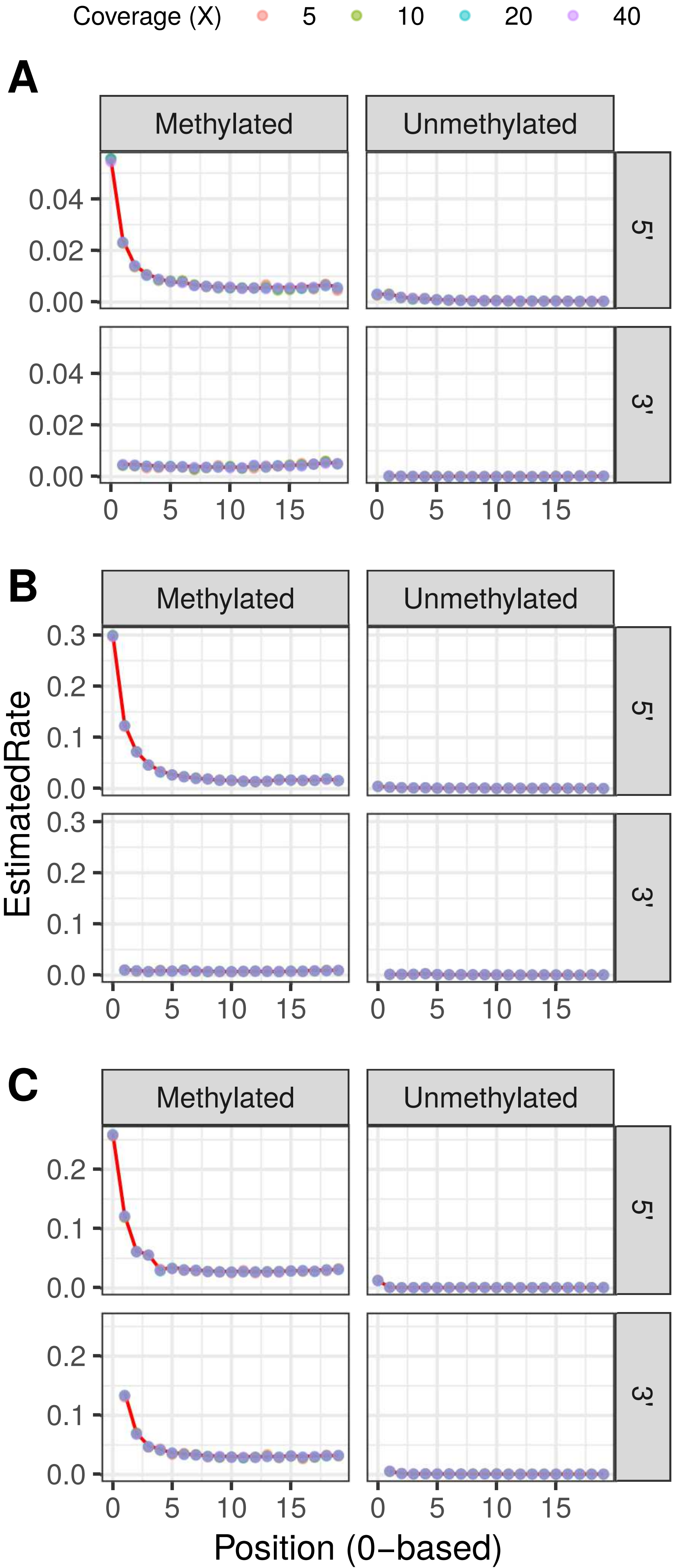

Figure 2

Click here to download Figure fig2.eps

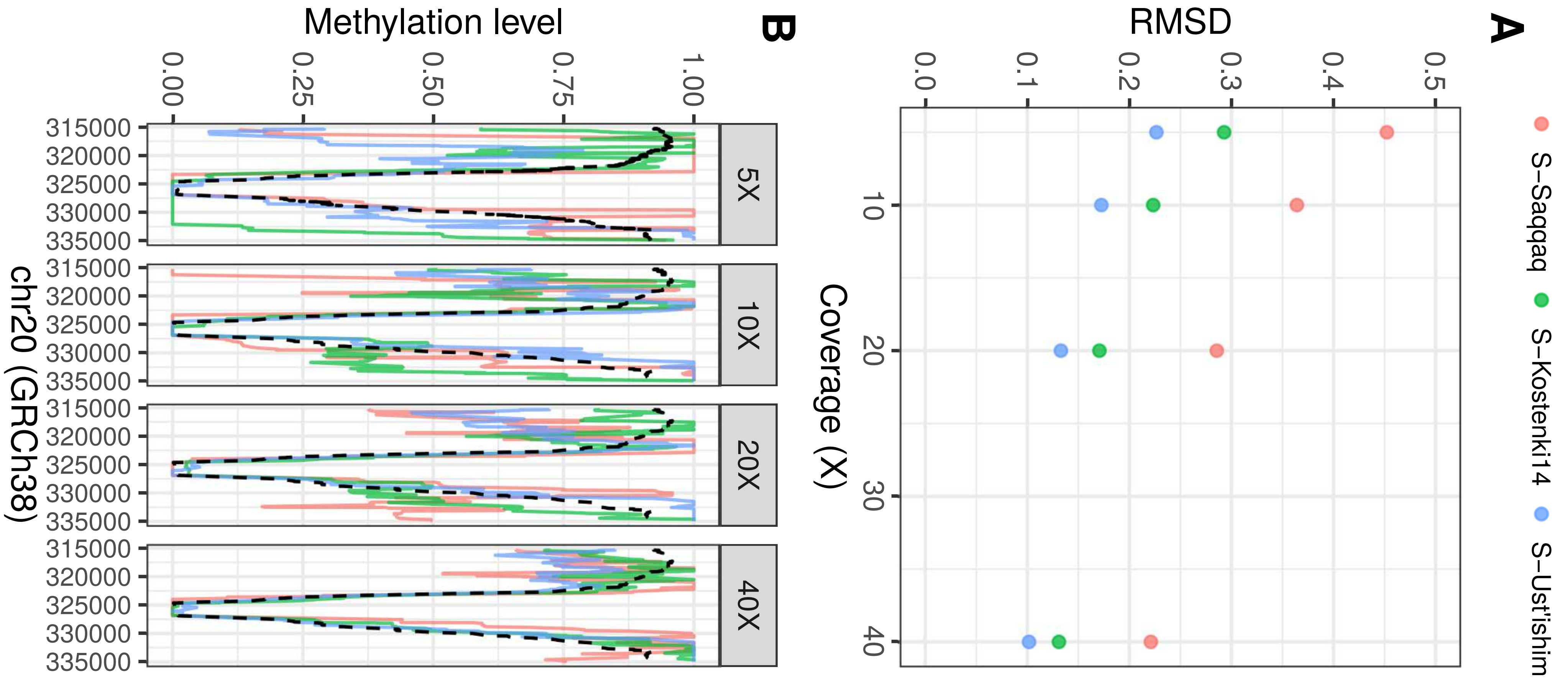

Figure 3

Click here to download Figure regional_methylation_main.eps

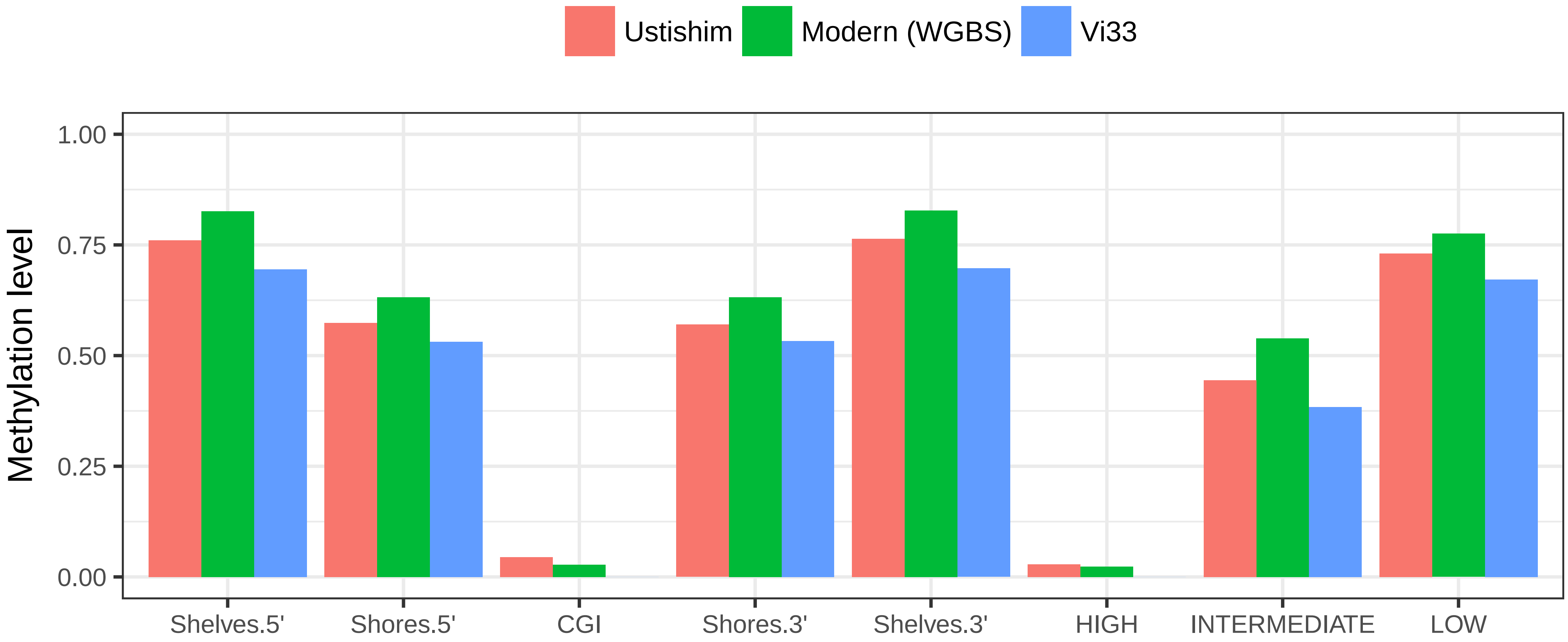

Supplementary information

Click here to access/download
Supplementary Material
supplementary.pdf

references

Click here to access/download
Supplementary Material
document.bib

Click here to access/download
Supplementary Material
gigascience-logo.pdf

Click here to access/download
Supplementary Material
main.bbl

Click here to access/download
Supplementary Material
oup-contemporary.cls

Click here to access/download
Supplementary Material
oup.pdf

Click here to access/download
Supplementary Material
regional_methylation_main_gigascience.eps

Click here to access/download
Supplementary Material
vancouver-authoryear.bst

Cover letter
Click here to download Personal Cover coverletter_KH_gigascience.docx

Dear Editor,

Please find enclosed our new manuscript "*DamMet, a full probabilistic model for mapping ancient methylomes*" by Hanghøj et al. We would like to publish this manuscript as an *Technical Note* in *GigaScience*. All co-authors have read and agreed on this version of the manuscript, including the companion software, DamMet. This package is implemented in C++ and can be found at *https://gitlab.com/KHanghoj/DamMet*, together with the necessary installation guide, manual, and tutorial.

DamMet is aimed at extracting DNA methylation information from the sequencing data underlying ancient genomes. This software will allow the community to easily track methylation changes through time from the hundreds of complete ancient genomes that are available today.

In contrast to current computational tools aimed at recovering ancient DNA methylation signals which are based on naïve mutational counts, DamMet is built around a full probabilistic model, that accounts for (1) the versatile properties of post-mortem DNA damage at both methylated and un-methylated CpG dinucleotide, (2) mapping and sequencing errors, and (3) genotyping variation. DamMet allows users to recover position-specific post-mortem cytosine deamination rates in both methylated and unmethylated contexts, which can be advantageously used to authenticate ancient DNA data. DamMet can then leverage such rates to provide Maximum Likelihood Estimates of regional methylation levels in any given genomic windows. These regional methylation levels are directly comparable between ancient methylomes and to modern methylomes.

We validate our approach using both simulated and real ancient DNA data, and describe a procedure allowing users, for the first time, to evaluate the amount of data required to obtain accurate estimates. This is implemented in gargammel (Renaud et al 2017), another software published by us.

Given the ongoing explosion of ancient DNA studies and the broad range of DamMet applications, we are convinced that DamMet will provide an important contribution to the fields of ancient DNA, evolutionary genomics and epigenomics. We would appreciate that Dr Liran Carmel, Dr David Gokhman and Dr Bastien Llamas are NOT considered as reviewers of the present work, due to likely conflict-of-interest. We would suggest Dr Robin Allaby, Dr Joachim Burger and Dr Laurent Frantz as potential reviewers.

I hope that you will consider our manuscript suitable for publication in GigaScience. Please do not hesitate to contact me, should anything remain unclear.

Sincerely,

Kristian Hanghøj
