# Supplementary material for: DamMet: ancient methylome mapping accounting for errors, true variants, and post-mortem DNA damage
Source: Gigascience. 2019 Apr 20;8(4):giz025. doi: 10.1093/gigascience/giz025 (PMC6474913; doi:10.1093/gigascience/giz025)
Supplement: Supplemental File [file giz025_supplemental_file.pdf]

Supplementary Material

for

DamMet: ancient methylome mapping accounting for errors, true variants and post-mortem DNA damage

Contents

1 Supplementary Methods **2**

1.1 Overall description of DamMet . . . 2

1.2 Step 1: Probabilistic model of position-specific post-mortem deamination at methylated and unmethylated cytosine residues . . . 3

1.2.1 Rationale . . . 3

1.2.2 Full likelihood model . . . 4

1.2.3 Obtaining a maximum likelihood estimate of $D_{M,k,v}$ . . . 5

1.3 Step 2: Full probabilistic model to recover regional methylation estimates . . . 5

1.3.1 Likelihood for regional window . . . 6

1.3.2 Maximum likelihood estimate of f . . . 8

1.4 DamMet, the software implementation . . . 8

2 Supplementary Results **9**

2.1 DamMet applied to simulated sequencing data . . . 9

2.1.1 Simulating ancient DNA sequencing data with methylation position-specific deamination patterns . . . 9

2.1.2 Three simulated scenarios . . . 10

2.1.3 Recovering post-mortem deamination rates . . . 11

2.1.4 Reconstruction of expected methylation profiles . . . 15

2.1.5 Recovering methylation profiles with high densities of true variants . . . 21

2.2 DamMet applied to sequencing data underlying ancient specimens 23

2.2.1 Identifying true variants in ancient genomes . . . 24

2.2.2 Reconstruction of deamination rates using real data . . . 25

2.2.3 Chromosome-wide methylation levels . . . 27

2.2.4 Validation of f using genomic regions with known methylation profiles . . . 28

1 Supplementary Methods

1.1 Overall description of DamMet

Recent computational advances in ancient DNA have shown that methylation profiles can be recovered from high-throughput DNA sequencing data by exploiting post-mortem deamination reactions in CpG contexts (Hanghøj *et al.*, 2016; Pedersen *et al.*, 2014; Gokhman *et al.*, 2014). In the recent years, two computational pipelines, epiPALEOMIX (Hanghøj *et al.*, 2016) and ROAM (Gokhman *et al.*, 2014), have been developed to map ancient CpG methylation profiles. Common to both pipelines is to consider all CpG sites present in a reference genome that are sequenced as TpG sites in an ancient individual as the hallmark of DNA methylation, given that (1) cytosine post-mortem deamination rates are faster at methylated sites than at un-methylated sites (Seguin-Orlando *et al.*, 2015; Smith *et al.*, 2015), and (2) that the molecular tools used for generating ancient DNA data at genome scales can significantly reduce or prevent sequencing unmethylated cytosines that deaminated into uracil residues (Briggs *et al.*, 2010). However, this approach has a number of caveats. Firstly, true sequence variants and sequencing errors can be found at CpG sites. Secondly, post-mortem DNA deamination reactions are not uniformly distributed along the DNA fragment and are inflated within overhanging ends (Briggs *et al.*, 2007); they can also show large variation in magnitude across DNA libraries, including from the same individual (Seguin-Orlando *et al.*, 2014). None such important characteristics are handled by the computational software currently available. Lastly, the available pipelines return a count statistic of the fraction of deamination events in a genomic window that require rescaling to be comparable to methylation data obtained with methodologies applied to modern samples. The transformation is usually achieved through statistical modeling, where the methylation scores measured in a modern methylome obtained from a given tissue are linearly correlated with those inferred from an ancient specimen (Gokhman *et al.*, 2014).

Here, we present DamMet, a new software to recover regional maximum likelihood estimates (MLE) of CpG methylation from sequencing data underlying an ancient specimen. DamMet tackles all limitations of the computational packages currently available for mapping ancient methylomes. In particular, DamMet relies on a new, more realistic statistical model of post-mortem DNA degradation at CpG sites, which integrates the actual deamination rates along a DNA fragment (per read group if needed), consider true variants in an unobserved dinucleotide genotype space, and handles sequencing errors. Lastly, MLE are directly comparable to that from modern methylation data, leaving no need for further statistical scaling of the count statistics provided.

The algorithm implemented in DamMet follows a two step procedure. The first step aims at obtaining MLE of the post-mortem deamination rates in a position-specific manner at both methylated and un-methylated cytosines. To disentangle deamination events of a methylated cytosines from unmethylated cytosines, we use a prior reflecting the expected fraction of methylated cytosines genome-wide. In mammals, the fraction of methylated cytosines in CpG context in somatic tissues is $70-80\%$ (Li and Zhang, 2014), meaning that $20-30\%$ are expected to be found in un-methylated states. The second step makes use of these deamination rates to recover a MLE of f, the fraction of methylated cells

in any given genomic window. A 95% confidence interval of f, is also generated. The software is freely available at https://gitlab.com/KHanghoj/DamMet.

1.2 Step 1: Probabilistic model of position-specific post-mortem deamination at methylated and unmethylated cytosine residues

The first step of the algorithm, is to recover absolute position-specific post-mortem deamination rates of methylated and unmethylated cytosine residues. In the following, we first describe why a single deamination parameter is insufficient. We then develop the model implemented in DamMet to obtain MLE of position-specific deamination rates.

1.2.1 Rationale

Position-specific deamination rates are pivotal due to an extremely uneven deamination rate along ancient DNA molecules. The latter often show overhanging ends (Briggs *et al.*, 2007), where post-mortem DNA degradation, in particular cytosine deamination, takes place up to 100 times faster than in double stranded context (Lindahl, 1993). It results in position-dependent post-mortem deamination rates, which inflate towards DNA fragment termini. Additionally, experimental evidence indicate that post-mortem cytosine deamination rates at methylated CpGs are faster than at un-methylated CpGs (Seguin-Orlando *et al.*, 2015; Smith *et al.*, 2015). Thus, using the same position-specific distribution of cytosine deamination rates for both methylated and unmethylated CpGs does not account for the real biochemical processes taking place after death.

The first step of the DamMet algorithm aims at providing MLE of post-mortem cytosine deamination rates in a position-specific manner at both methylated and un-methylated CpGs. These distributions are exploited in the second step to derive accurate estimates of the methylation levels of any given (and user-defined) genomic window. More specifically, we estimate the methylated ($D_{M=1}$) and unmethylated ($D_{M=0}$) deamination rates per DNA fragment position (k) from either the 5' or the 3' end ($v \in (5', 3')$) of the DNA molecule using all the available sequencing data per chromosome. Our approach assigns v to the prime closest to position k of the DNA molecule, if equidistant $v = 5'$ is assigned. The number of positions k for which deamination rates are estimated are user-defined. The default number of positions are 30 per prime. We estimate the deamination rates per chromosome, rather than globally, to limit the possible impact of differences in the quality of individual chromosomal assemblies on downstream analyses. The rates can also be estimated per read group. Finally, the model accounts for sequencing errors and mapping errors. However, to reduce noise from sequencing errors in the estimates of the deamination rates, we disregard nucleotides with a PHRED quality score < 30. We also define a global prior on the total fraction of methylated CpGs (F_{global}) present in the genome. This prior is by default adjusted for mammals to 75% based on empirical data (Li and Zhang, 2014), but can be changed if necessary. Additionally, cytosines present in a CpG context (CpGs) and cytosines not included within CpGs (CnonCpGs) are considered together in the full likelihood, using equal number of observations for both categories. By including CnonCpGs data observations, we improve the statistical power for recovering optimal deamination

rates for unmethylated cytosine residues.

1.2.2 Full likelihood model

The full likelihood of the position-specific deamination rates ($D_{M,k,v}$) is based on chromosome-wide DNA fragment observations ($\mathbb{D}$) covering a cytosine in the reference genome, including all observations with a cytosine in a CpG context and an equal number of CnonCpGs. We assume independence between DNA fragment observations and between sites along the chromosome.

We define $L(D|\mathbb{D})$, the likelihood of position-specific post-mortem cytosine deamination rates given chromosome-wide sequencing data, as follows:

$$L(D|\mathbb{D}) = \prod_{j=1}^{sites} \prod_{i=1}^{depth} p(X_{j,i,k,v}|D_{M,k,v}, \epsilon_{j,i}, Q_{j,i}, F_{global}) \tag{1}$$

, where $D_{M,k,v}$ denotes the current estimate of post-mortem cytosine deamination at DNA fragment position k prime v of a DNA fragment, within a methylated context ($M = 1$) and an unmethylated context ($M = 0$). Additionally, $\epsilon_{j,i}$ is the probability of a sequencing error, obtained from the base calling, at observation $X_{j,i,k,v}$, $Q_{j,i}$ the probability of a mapping error, obtained from the read-mapper, for the DNA fragment (i) considered at site (j), and F_{global} is the user defined overall fraction of methylated cytosines. The probability of an observation $X_{j,i,k,v}$ at position k from prime v of a DNA molecule given a cytosine in the reference ($B = C_{CpG}$), the position-specific deamination rate ($D_{M,k,v}$), and the probability of a mapping error is the following:

$$\begin{aligned}
&p(X_{j,i,k,v}|D_{M,k,v}, \epsilon_{j,i}, Q_{j,i}, F_{global}) = \\
&(1 - Q_{j,i})\big(\\
&F_{global}p(X_{j,i,k,v}|D_{M=1,k,v}, B = C_{CpG}, \epsilon_{j,i})+ \\
&(1 - F_{global})p(X_{j,i,k,v}|D_{M=0,kv}, B = C_{CpG}, \epsilon_{j,i}) \\
&\big) + Q_{j,i}P_x
\end{aligned} \tag{2}$$

, where $P_x = 0.25$ is the prior probability of observing any nucleotide. For DNA fragment observations not located in a CpG context ($B = C_{CnonCpG}$), the model assumes $F_{global} = 0$, thus eq. 2 simplifies to

$$\begin{aligned}
&p(X_{j,i,k,v}|D_{M=0,k,v}, \epsilon_{j,i}, Q_{j,i}) = \\
&(1 - Q_{j,i})p(X_{j,i,k,v}|D_{M=0,k,v}, B = C_{CnonCpG}, \epsilon_{j,i}) + Q_{j,i}P_x
\end{aligned} \tag{3}$$

. The probability $p(X_{j,i,k,v}|D_M, B = C, \epsilon)$ has the following possible outcomes:

$$\begin{aligned}
&p(X_{j,i,k,v}|D_{M\in(0,1),k,v}, B = C, \epsilon_{j,i}) = \\
&\begin{cases}
(1 - \epsilon_{j,i})(1 - D_{M,k,v}) + D_{M,k,v}\frac{\epsilon_{j,i}}{3} + \epsilon_{j,i}\frac{\epsilon_{j,i}}{3} & \text{if } X_{j,i,k,v} = C \\
(1 - \epsilon_{j,i})D_{M,k,v} + (1 - D_{M,k,v})\frac{\epsilon_{j,i}}{3} + \epsilon_{j,i}\frac{\epsilon_{j,i}}{3} & \text{if } X_{j,i,k,v} = T \\
(1 - D_{M,k,v})\frac{\epsilon_{j,i}}{3} + D_{M,k,v}\frac{\epsilon_{j,i}}{3} + \frac{\epsilon_{j,i}}{3} & \text{if } X_{j,i,k,v} \in (A, G)
\end{cases}
\end{aligned} \tag{4}$$

. The first outcome in equation 4 corresponds to observing a cytosine residue ($X_{i,k,v} = C$) covering a cytosine in the reference genome located in either in a

CpG or nonCpG context. We consider three possible probabilities for which this can occur, (i) no sequencing error $(1-\epsilon_i)$ and no deamination event $(1-D_{M,k,v})$, (ii) a deamination event occurred $(D_{M,k,v})$ followed by type specific a sequencing error to a cytosine residue $(\frac{\epsilon_i}{3})$, (iii) a sequencing error occurred (ϵ_i) follow by a type specific a sequencing error to a cytosine residue $(\frac{\epsilon_i}{3})$. The second and third outcome of equation 4 describe the three possibilities for observing a thymine residue $(X_{i,k,v}) = T$ and a purine $(X_{i,k,v}) \in (A, G)$, respectively, when covering a cytosine in the reference genome.

To improve the readability of equations 1-4, we have shown the method and notations for DNA fragments aligned to the positive strand only. DamMet, however, takes advantage of the whole amount of sequence data both those aligned against the positive and the negative strands. The model is shown in natural space, however, the implementation is done in natural-log space to avoid numerical overflow.

1.2.3 Obtaining a maximum likelihood estimate of $D_{M,k,v}$

By maximizing the full likelihood function described in section 1.2.2, we can obtain a MLE of the position-specific deamination rates $(D_{M,k,v})$ both at methylated and in unmethylated cytosines

$$\hat{D}_{M,k,v} = argmax(L(D_{M,k,v}|\mathbb{D})) \tag{5}$$

. To obtain the MLE of the position-specific deamination rates $(D_{M,k,v})$, we use a derived-based $(\frac{\partial L(D_{M,k,v}|\mathbb{D})}{\partial D_{M,k,v}})$ gradient descent optimization method named MMA (Svanberg, 2002) as implemented in C++ in nlopt library.

1.3 Step 2: Full probabilistic model to recover regional methylation estimates

In this section, we describe the second step of the model implemented in DamMet, which relies on a full likelihood function to recover maximum likelihood estimates of f, the fraction of methylated cells in a user-defined genomic window. The likelihood includes all observed sequencing data covering CpGs in a user-defined genomic (S) window and the model parameter of interest f. The model accounts for the uncertainty of sequencing errors (ϵ), mapping errors (Q_i), and importantly the probability of the presence of true variants (G) (fig. 1). To incorporate the latter, we sum over a subset of possible dinucleotide genotypes, where each dinucleotide genotype $G = g$ consists of two independent genotypes at both positions of the dinucleotide $(g_{j=1}, g_{j=2})$ as shown in fig.1. We have reduced the set of true possible dinucleotide genotypes in our model from 256 to the only seven that can possibly confuse methylation signatures at CpG sites. This considerably reduces the computational cost. The seven dinucleotide genotypes have at most a single alternative allele per dinucleotide genotype, thus, assume no double mutations. Finally, we assume that methylation of cytosines cannot co-occur together with an alternative allele. As a result, methylation can only be observed in one of the seven dinucleotide genotypes considered, namely $G = 0$. The last parameter used by this model is the position-specific deamination rates of methylated and unmethylated cytosines ($D_{M,k,v}$ hereafter referred to as D_M to improve readability) estimated in step one (section 1.2). The

model assumes that all DNA fragments are mapped to the positive strand, thus methylation of cytosine on the negative strand in a CpG context is reflected in the guanine residue on the positive strand. The model cannot distinguish methylation levels for the cytosine on the positive and negative strand in a CpG context, individually. For every estimate, the 95% confidence interval of f is calculated using the second derivative of the full likelihood function.

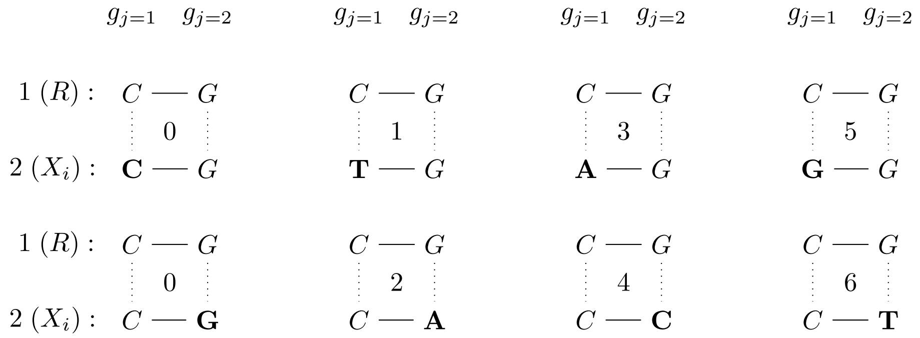

Figure 1: Dinucleotide genotypes considered. Dinucleotides are connected by solid lines, genotypes ($g_{j\in(1,2)}$) by dashed lines. Dinucleotides are always consecutive nucleotides on a DNA fragment. R is the dinucleotide present in the reference genome, X_i is the ith dinucleotide from the pile of sequencing data ($X_{i,j\in(1,2)}$). The seven different dinucleotide genotypes each have a reference number from 0 to 6. Dinucleotide genotype 0, homozygous cytosine genotype followed by homozyogus guanine genotype, is present twice for illustrative purposes.

1.3.1 Likelihood for regional window

We calculate the likelihood function of the model in a genomic window containing a set of CpGs (S), incorporating all sequencing data overlapping these CpGs ($\mathbb{D}$) and the parameter of interest f. X is our dinucleotide sequencing data observations per site s. Each dinucleotide observation (X_i) consists of two nucleotides, namely ($X_{i,j=1}$) and ($X_{i,j=2}$) with individual probabilities of a sequencing error ϵ_j and the probability of a mapping error Q_i for DNA fragment i. Likewise, our set of unobserved dinucleotide genotypes ($G = g$) consists of two genotypes ($G = g_{j=1}$) and ($G = g_{j=2}$) (fig: 1). The likelihood function of the fraction of cells showing methylated states at the considered genomic windows given the data is:

$$L(f|\mathbb{D}) = \prod_{s}^{sites} \sum_{G\in(0...6)} p(G = g)p(X|s, f, G = g, D, \theta) \tag{6}$$

,

where $p(X|s, f, G = g, D, \theta)$ is the probability of the data at a site s given an unobserved dinucleotide genotype g. $p(G = g)$ is the prior probability of the unobserved dinucleotide genotype. Assuming independence between DNA fragment observations (X) and between the two genotypes per dinucleotide

genotype $G = g$, we get for all genotypes where methylation cannot take place ($G \in (1 \dots 6)$) the following probability of the data for a single dinucleotide genotype,

$$
\begin{aligned}
&p(X|s, f = 0, G, D, \theta) = \\
&\prod_{i=1}^{depth} (1 - Q_i)\big(p(X_{ij=1}|D_M, G = g_{j=1}, \epsilon_{j=1}, f = 0) \\
&\quad p(X_{ij=2}|D_M, G = g_{j=2}, \epsilon_{j=2}, f = 0)\big) + \\
&Q_i P_g
\end{aligned}
\tag{7}
$$

, where $P_g = \frac{1}{7}$ denotes a prior that all seven dinucleotide genotypes considered are equally likely and D_M denotes the MLE of the position-specific deamination rates estimated in step one (section 1.2).

Equation 7 is applied to dinucleotide genotypes $G \in (1 \dots 6)$ (see fig. 1) but must be extended to the following for $G = 0$, given that methylation can only take place within this context:

$$
\begin{aligned}
&p(X|f, G_{g=0}, D, \theta) = \\
&\prod_N (1 - Q_i)\Big(\\
&f\big(p(X_{ij=1}|D_{M=1}, G = g_{j=1}, \epsilon_{j=1})p(X_{ij=2}|D_{M=1}, G = g_{j=2}, \epsilon_{j=2})\big) + \\
&(1 - f)\big(p(X_{ij=1}|D_{M=0}, G = g_{j=1}, \epsilon_{j=1})p(X_{ij=2}|D_{M=0}, G = g_{j=2}, \epsilon_{j=2})\big) \\
&\Big) + Q_i P_g
\end{aligned}
\tag{8}
$$

.

The probability of an observation ($X_{ij} = \{A, C, G, T\}$) given, position-specific deamination rates, an unobserved genotype ($g_j = (B_1, B_2)$), and the probability of a sequencing error (ϵ) is

$$
P(X_{ij}|D_M, g_j = (B_1, B_2), \epsilon) = (\frac{1}{2}p(X_{ij}|D_M, B_1, \epsilon) + \frac{1}{2}p(X_{ij}|D_M, B_2, \epsilon)) \tag{9}
$$

, where the observation X_i can take at most take of one of the two alleles (B_1, B_2). The model assumes that the unobserved alleles are equally likely.

An observation X_{ij} has three possible outcomes, when the unobserved allele is a cytosine (C).

$$
\begin{aligned}
&p(X_{ij}|D_{M\in(0,1)}, B = C, \epsilon) = \\
&\begin{cases}
(1 - \epsilon)(1 - D_M) + D_M\frac{\epsilon}{3} + \epsilon\frac{\epsilon}{3} & \text{if } X_{ij} = C \\
(1 - \epsilon)D_M + (1 - D_M)\frac{\epsilon}{3} + \epsilon\frac{\epsilon}{3} & \text{if } X_{ij} = T \\
(1 - D_M)\frac{\epsilon}{3} + D_M\frac{\epsilon}{3} + \frac{\epsilon}{3} & \text{if } X_{ij} \in (A, G)
\end{cases}
\end{aligned}
\tag{10}
$$

.

When $B \neq C$, eq. 10 simplifies to,

$$
p(X_{ij}|B \neq C, \epsilon) = \begin{cases}
(1 - \epsilon) & \text{if } X_{ij} = g \\
\frac{\epsilon}{3} & \text{if } X_{ij} \neq g
\end{cases}
\tag{11}
$$

, as deamination can only modify cytosine residues.

1.3.2 Maximum likelihood estimate of f

$$\hat{f} = argmax(L(f|\mathbb{D})) \tag{12}$$

. To get the maximum likelihood estimate of the regional f, we use the derived-based $\frac{\partial l(f,\mathbb{D})}{\partial f}$ gradient descent optimization method MMA (Svanberg, 2002) as implemented in nlopt. DamMet also provides 95% confidence intervals of f using the second derivative of the maximum likelihood function,

$$\frac{1.96}{\sqrt{-\frac{\partial^2 l(\hat{f},\mathbb{D})}{\partial^2 \hat{f}}}} \tag{13}$$

.

The MLE of f is a continuous variable ranging from $1e^{-7}$ to 1. Importantly, the estimate is directly comparable to methylation data obtained from modern methodologies such as WGBS data.

1.4 DamMet, the software implementation

The models described above are implemented in DamMet in C++ and optimizations are done with nlopt also implemented in C++. BAM files are parsed with htslib. DamMet can be installed with the following commands

Installation

```
git clone https://gitlab.com/KHanghoj/DamMet.git
cd DamMet && make && cd ..
```

Running example of DamMet

Below is a brief tutorial including a running example. The input data, 'test.bam' in DamMet-tutorial.git, is a sliced (chr20:315000-335000; GRCh38) version of the S-Kostenki14 simulated sample with 30-fold coverage. In this example, we recover the sample profile as shown in fig 11. We are using pre-calculated deamination rates, as the sliced input data ('test.bam') contains too few data points to get confident estimates. The output, 'result.pdf', contains both the expected methylation profile (red line) and the estimates of f (black circles).

```
git clone https://gitlab.com/KHanghoj/DamMet-tutorial.git
cd DamMet-tutorial
git clone https://gitlab.com/KHanghoj/DamMet.git
cd DamMet && make && cd ..
bash run.sh
```

See https://gitlab.com/KHanghoj/DamMet for an in-depth description of the available arguments for DamMet and of its output format.

2 Supplementary Results

2.1 DamMet applied to simulated sequencing data

2.1.1 Simulating ancient DNA sequencing data with methylation position-specific deamination patterns

In order to test the two models implemented in DamMet, we should apply the methodology to simulated data where the results are known a priori. However, the only read simulator currently available for ancient DNA data, gargammel (Renaud *et al.*, 2017), does not take DNA methylation patterns into account. We thus developed the first simulator to generate ancient DNA sequence data with methylation and position-specific deamination patterns. The procedure, implemented as a new feature in gargammel (Renaud *et al.*, 2017), is briefly described below.

First, we generate a diploid genome, using a reference genome and VCF file (SS6004469) providing information about the location and nature of polymorphic sites. This diploid genome is copied 100 times, as to represent the diploid genomes of 100 cells. Using the methylation value (M) empirically estimated from methylation data, M cells per CpG are marked as methylated and 100-M cells as unmethylated. Any input data, in bedMethyl format, can be used for methylation scores. We have used ENCFF103DNU WGBS methylome from the ENCODE project to apply methylation marks for every cell at every CpG in this study. In the preprocessing of the WGBS BED file, we merged the methylation scores for each strand of each CpG. This is required as the simulator identifies a CpG genomic site as a single unit with equal probability of deaminating the cytosine residue on each strand. CpG sites covered by less than ten reads in the WGBS BED file are masked from any downstream analyses. Methylation marks can only be applied to CpG's without any alternative alleles in the dinucleotide genotype. Next, the diploid cells are split into two haploid genomes, retaining their methylation marks. We then sample sequence data with replacement, from the cells and apply the position-specific deamination matrix based on the methylation status ($M \in (0, 1)$) of the cytosines across every sampled sequence from every cell. Cytosines found outside a CpG context (CnonCpG) are assumed to deaminate with the same probability as unmethylated cytosines located in a CpG context. Lastly adapters are added as described in the original publication (Renaud *et al.*, 2017). See Figure 2 for a flowchart of the new feature implemented in gargammel. gargammel is freely available at https://github.com/grenaud/gargammel.

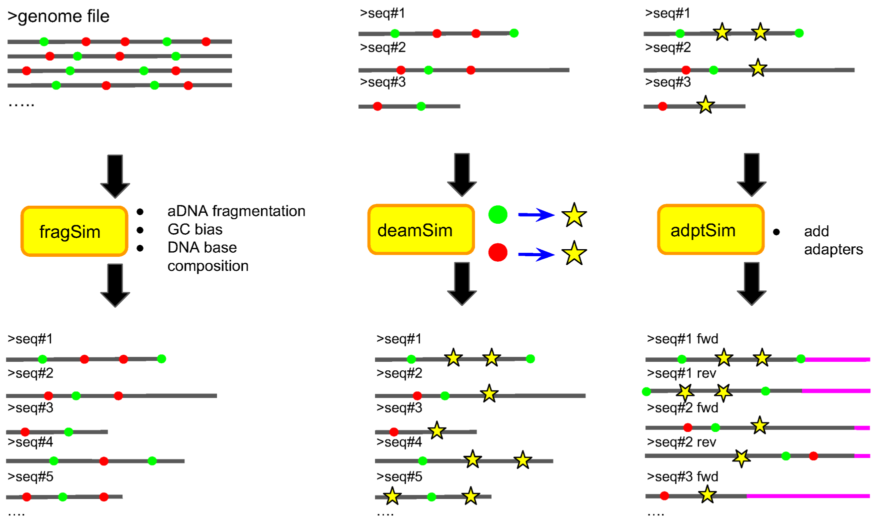

Figure 2: Flowchart for the 3 main sub-programs in the gargammel (Renaud *et al.*, 2017) modified to illustrate the addition of methylated cytosine residues (red) and unmethylated cytosine residue (green). fragSim sample DNA molecules from the 100 cells. deamSim adds deamination events of both methylated and unmethylated cytosines. After adptSim adds adapters (magenta) to the DNA molecules, the final reads are then generated with ART (Huang *et al.*, 2012). Stars illustrate deamination events.

2.1.2 Three simulated scenarios

In this study, we simulated sequencing data using the position-specific deamination profiles obtained form three different samples, namely Saqqaq (Rasmussen *et al.*, 2010), Kostenki14 (Seguin-Orlando *et al.*, 2014), and Ust'ishim (hereafter referred to as Ustishim) (Fu *et al.*, 2014).

These three samples exhibit a whole range of possible deamination rates and they represent two main types of library protocols most often used in ancient DNA research (the double stranded (Saqqaq, Kostenki14) and single stranded (Ustishim) library protocol). Ustishim and Kostenki14 DNA extracts both underwent a USER treatment (Briggs *et al.*, 2010) prior to library building. This treatment excises almost all uracil residues in the sequencing data that are a result of deamination events of an unmethylated cytosine residues. The DNA extract of the saqqaq sample did not undergo a USER treatment, however, DNA library templates including uracil residues could not be sequenced given the molecular tools used (Pedersen *et al.*, 2014), resulting in similar outcomes. To avoid naming confusion, we have added a 'S-' prefix to the original sample name to illustrate that the sequencing data are simulated.

In all three cases, we have used the same read length distribution for the sampling of DNA molecules, the one found in the sequencing data of the Ustishim sample. This makes the accuracy of DamMet to estimate both position-specific deamination profiles (D_M) and the local estimate of methylated cells (f), comparable across samples. For every scenario, we simulated sequencing data as

described in section 2.1.1 from chromosome 20 (GRCh38) and mapped with BWA backtrack (Li and Durbin, 2009) (default settings; seed disabled) to obtain a 100-fold coverage alignment file (BAM file). Next, the BAM files were downsampled to cover the whole range of observed coverage identified in ancient DNA studies down to 5-fold coverage. For the remaining analyses based on simulated data, we have used this set of BAM files. All simulated BAM files can be downloaded from ERDA, an electronic scientific data repository provided and maintained by the University of Copenhagen (link).

2.1.3 Recovering post-mortem deamination rates

The first step in the algorithm implemented in DamMet is to obtain maximum likelihood estimates (eq. 5) of position-specific post-mortem deamination rates of methylated and unmethylated cytosines. With the simulated data generated as described in section 2.1.1 using three scenarios (section 2.1.2), we can test the accuracy and robustness of the maximum likelihood estimates provided by our methodology.

We find that DamMet recovers position-specific deamination estimates highly similar to those expected in all three scenarios (fig 3,4,5). However, we see a slight underestimate of the deamination rates of methylated cytosines in the 5' when using the simulated sequencing data with a minimum length of 25bp mapping to the reference genome. This slight underestimate likely originates from an excess of mismatches due to the extreme rate of deamination of methylated cytosines in the first DNA fragment positions, leaving, predominantly, short DNA molecules with a too-high edit distance to the reference to be unmappable with BWA backtrack (Li and Durbin, 2009) (default settings; seed disabled). Restricting the estimates to DNA fragments >=60 (-l 60), we recover almost exact deamination rates (fig 3,4,5). We also investigated the RMSD of the position-specific deamination rates as a function of coverage (X) and minimum DNA fragment length (fig 6). Consistently, the overall estimate is more accurate using DNA fragments equal to or longer than 60bp. We also find a slightly elevated deviation at a sequencing depth of 5X when using simulated data exclusively from chromosome 20 (approximately 60 million bp). Estimating the deamination rates from a larger chromosome improves the accuracy notably (data not shown).

Important to note, increasing the minimum DNA fragment length to 60bp for estimating the deamination parameters in this example is not a general recommendation, given that ancient DNA data generally consist of DNA fragments shorter than this size threshold (hence, the threshold should be selected by the users on a DNA library and/or sample basis). It was solely done here to show that the underestimate identified when including very short DNA fragments was due to a mapping bias and not a bias in the DamMet algorithm.

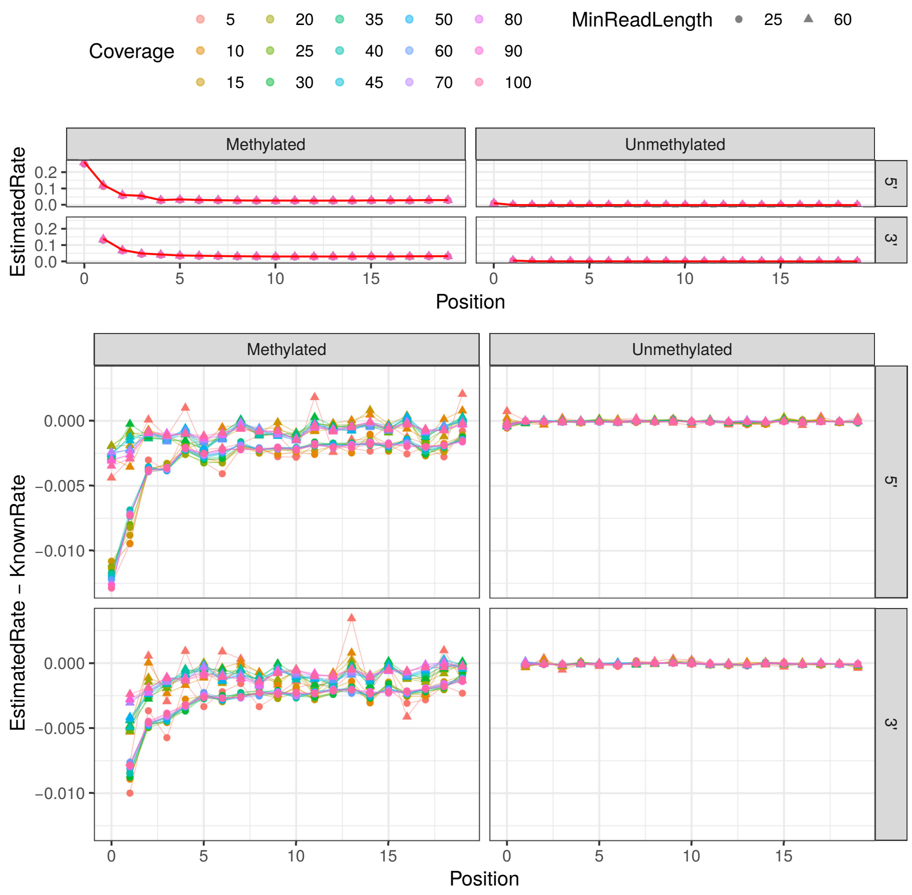

Figure 3: Estimated deamination rates, from simulated sequencing data of S-Ustishim, of (un)methylated cytosines for the first 20 positions of the 5' and 3' of termini a DNA molecule. Estimates are shown with a minimum readlength of 25 and 60 (MinReadLength) across a range of possible coverages (X-fold). Top panel: Known deamination rates are shown as a red line. Lower panel: Accuracy in the form of Estimated deamination rates - Known deamination rates.

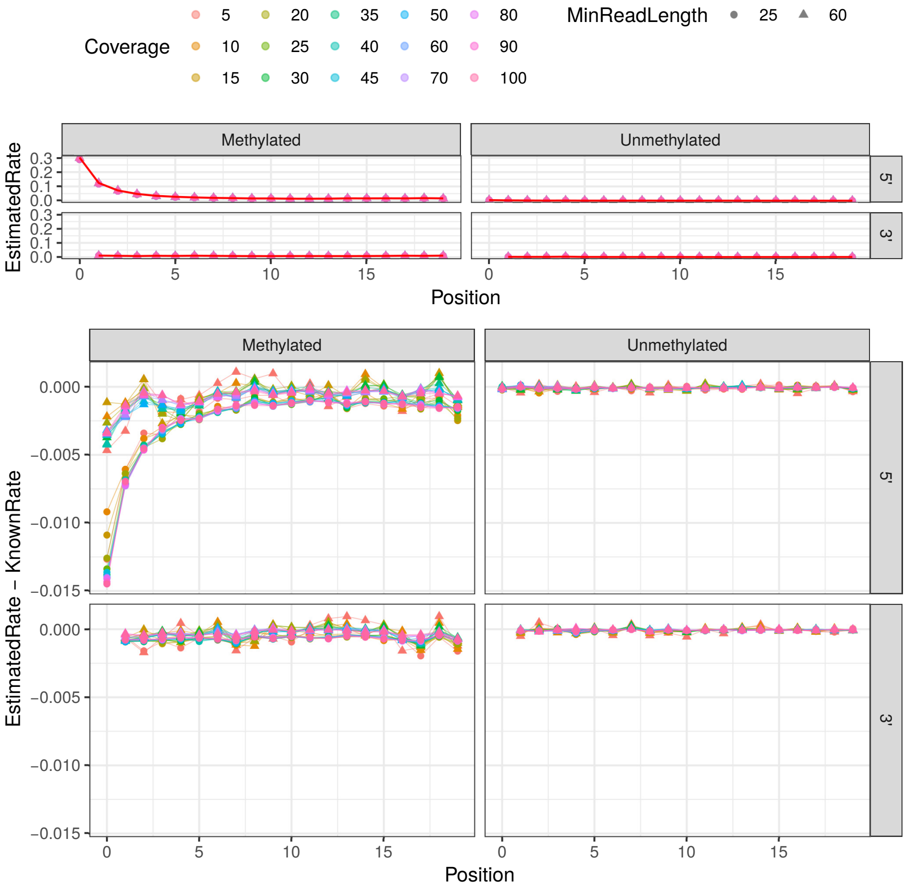

Figure 4: Estimated deamination rates, from simulated sequencing data of S-Kostenki14, of (un)methylated cytosines for the first 20 positions of the 5' and 3' of termini a DNA molecule. Estimates are shown with a minimum readlength of 25 and 60 (MinReadLength) across a range of possible coverages (X-fold). Top panel: Known deamination rates are shown as a red line. Lower panel: Accuracy in the form of Estimated deamination rates - Known deamination rates.

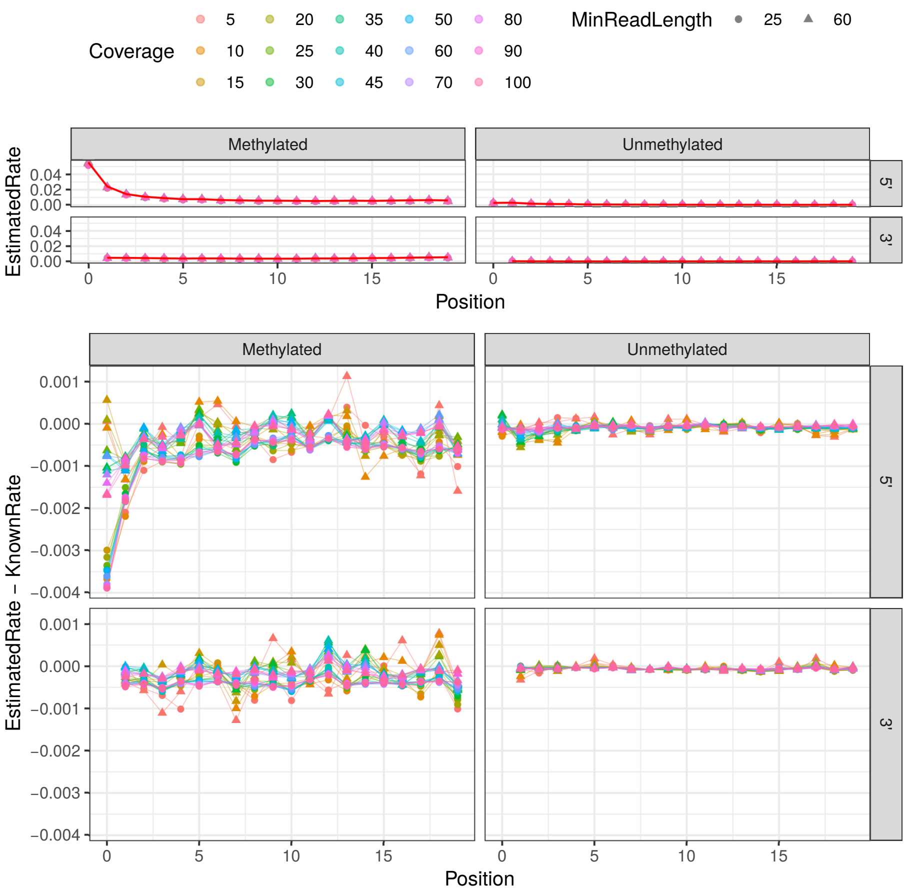

Figure 5: Estimated deamination rates, from simulated sequencing data of S-Saqqaq, of (un)methylated cytosines for the first 20 positions of the 5' and 3' of termini a DNA molecule. Estimates are shown with a minimum readlength of 25 and 60 (MinReadLength) across a range of possible coverages (X-fold). Top panel: Known deamination rates are shown as a red line. Lower panel: Accuracy in the form of Estimated deamination rates - Known deamination rates.

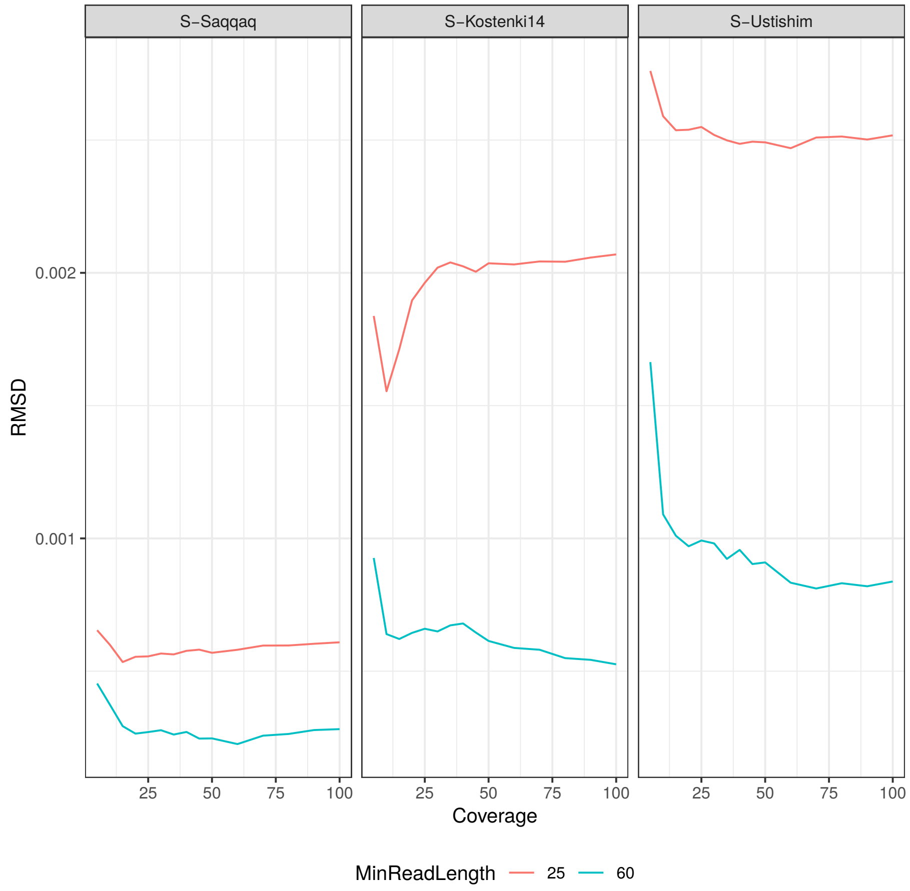

Figure 6: Root mean square deviation (RMSD) of the estimated deamination rates shown in figure 3-5.

2.1.4 Reconstruction of expected methylation profiles

The second step in the model implemented in DamMet is to obtain a maximum likelihood estimate of f, the fraction of methylated cells in a genomic region. We assessed the accuracy of DamMet using the three simulated datasets described in section 2.1.2.

Chromosome wide accuracy of f. First, we investigated how well DamMet recovers chromosome-wide estimates of f as a function of sequencing efforts (Coverage X) and window size, as measured as number of CpGs per window (N_CpG).

In Figure 7, we confirm the expectation that the root mean squared deviation (RMSD) of the f estimate, decreases with increasing sequencing effort and window sizes for all tested scenarios. We also consistently find the highest accuracy (lowest RMSD) for S-Ustishim, followed by S-Kostenki14 and S-Saqqaq. This order is, as expected, inversely correlated with the magnitude of the post-

mortem deamination rates measured in all three samples (which provide the source for the methylation signature).

We also investigated the distribution of the accuracy, in the form of the difference between the estimate and the known value of f (Estimated_f - Known_methylation), per simulated scenario for every combination of window size and depth-of-coverage (fig 8,9,10). We find that the accuracy is symmetrically centered around zero, with slightly underestimated values for smaller genomic windows and/or limited sequencing effort. These minor underestimates are a result of low statistical power, due to the absence of sampled deamination events when coverage/window size is too low.

For small windows (N_CpG<20) and a depth-of-coverage $<$ 20-fold, we find a surprisingly large variance in accuracy regardless of the deamination profiles used in the simulations. These results suggest that single nucleotide resolution methylation maps could probably not be achieved with high accuracy using current methodological approaches, including for 100-fold genome data and deamination rates similar to those measured in specimens showing similar degradation as Ustishim.

Nonetheless, these results demonstrate that DamMet recovers the exact estimate of f with sufficient sequencing effort and/or window size. This finding also illustrates the power of combining DamMet with the new simulator implemented in gargammel, as it offers the first opportunity to make qualified decisions on the amount of sequencing effort needed to obtain a given accuracy in the estimates of f for any particular sample/library. This can prove instrumental when deciding which samples should be deep sequenced or for undertaking the necessary power studies underlying the design of new research projects.

Local estimates of f. We also investigated how f resembles the expected regional methylation profile across a small genomic window (chr20:315000-335000; GRCh38). This region contains a 3437 bp long CpG island (chr20:324243-327679) with shores and shelves. We know on the basis of the simulated data (black dashed line), that this region forms a bell shaped methylation profile centered around the hypomethylated CpG island. The methylation level gradually increases as a function of distance from the CpG island. As shown in figure 11, DamMet can recover the bell shaped curved quite accurately, when sequencing effort and/or genomic window sizes are sufficiently large. DamMet can even retrieve the drop in methylation at the CpG island even at 10-fold coverage and a dynamic window size of 30 CpGs, however, the estimates of f surrounding the CpG island is still quite fluctuating with these experimental conditions. It is important to stress that the optimal trade off between resolution and the window size and/or sequencing effort should be decided by the user preferably based on simulations using the new simulator accompanying DamMet. Thus, no general guidelines can be provided as the power achieved depends directly on the post-mortem DNA degradation levels observed in each individual sample/library.

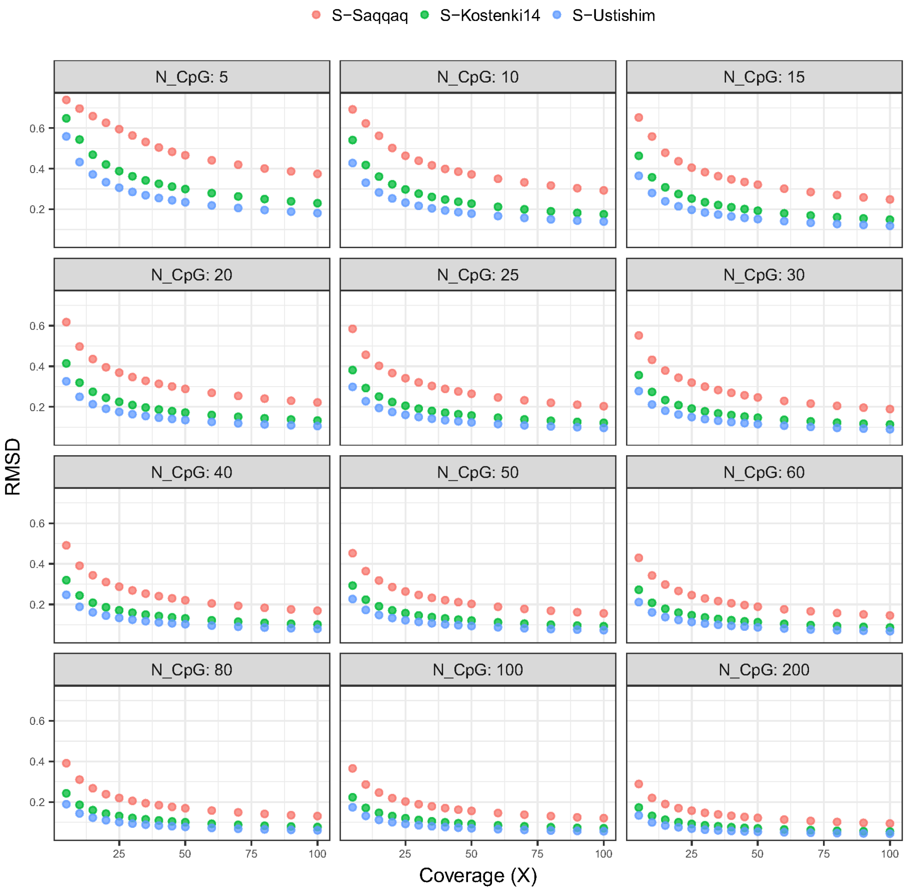

Figure 7: RMSD of f estimates (Estimated_f - Known_methylation) for a range of window sizes (measured as number of CpGs) for the three simulated datasets

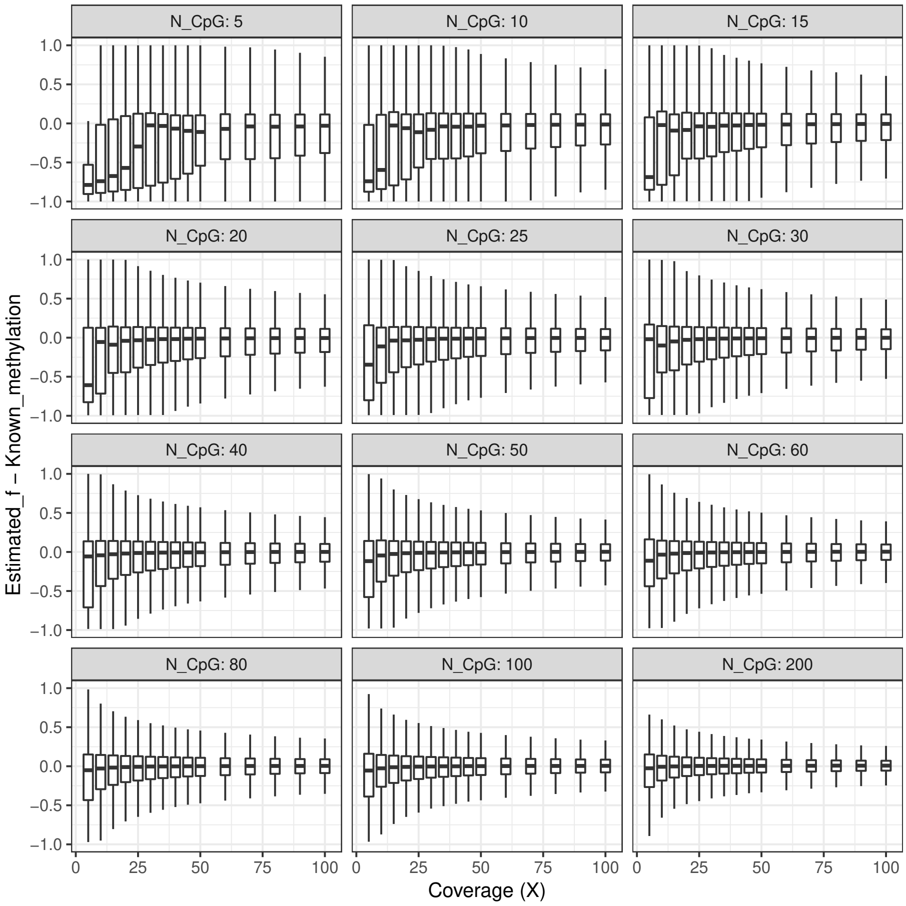

Figure 8: Boxplot of the accuracy of f (Estimated_f - known methylation) using the data from S-Saqqaq

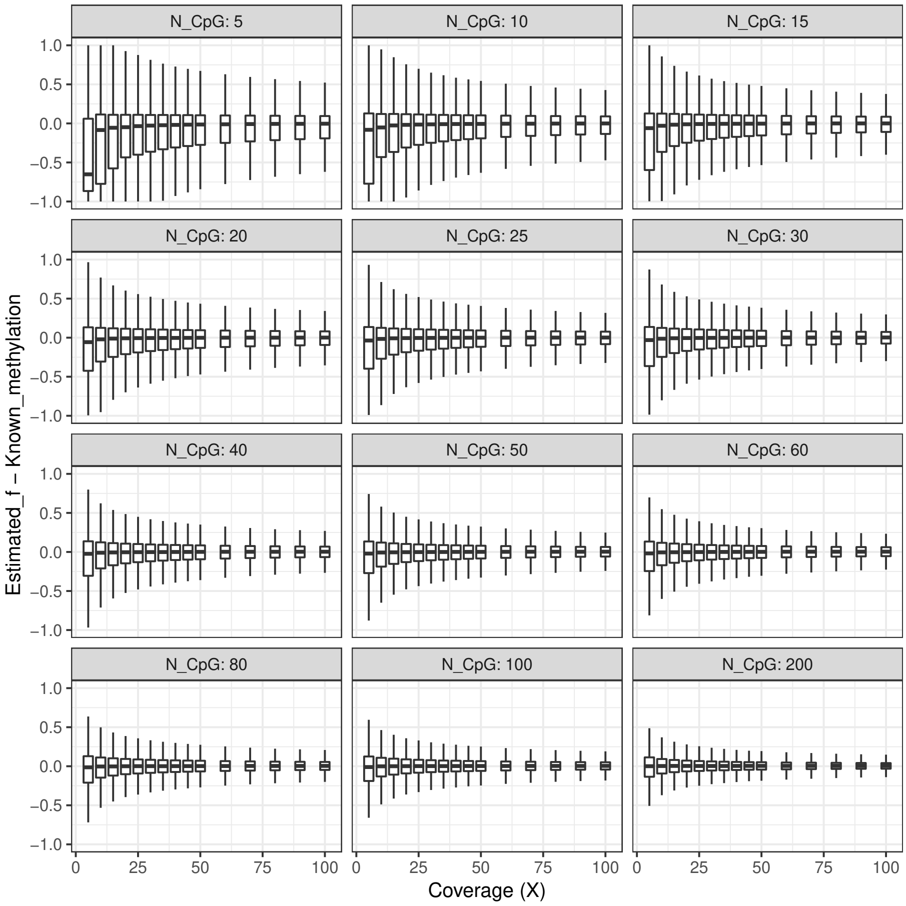

Figure 9: Boxplot of the accuracy of f (Estimated_f - known methylation) using the data from S-Kostenki14

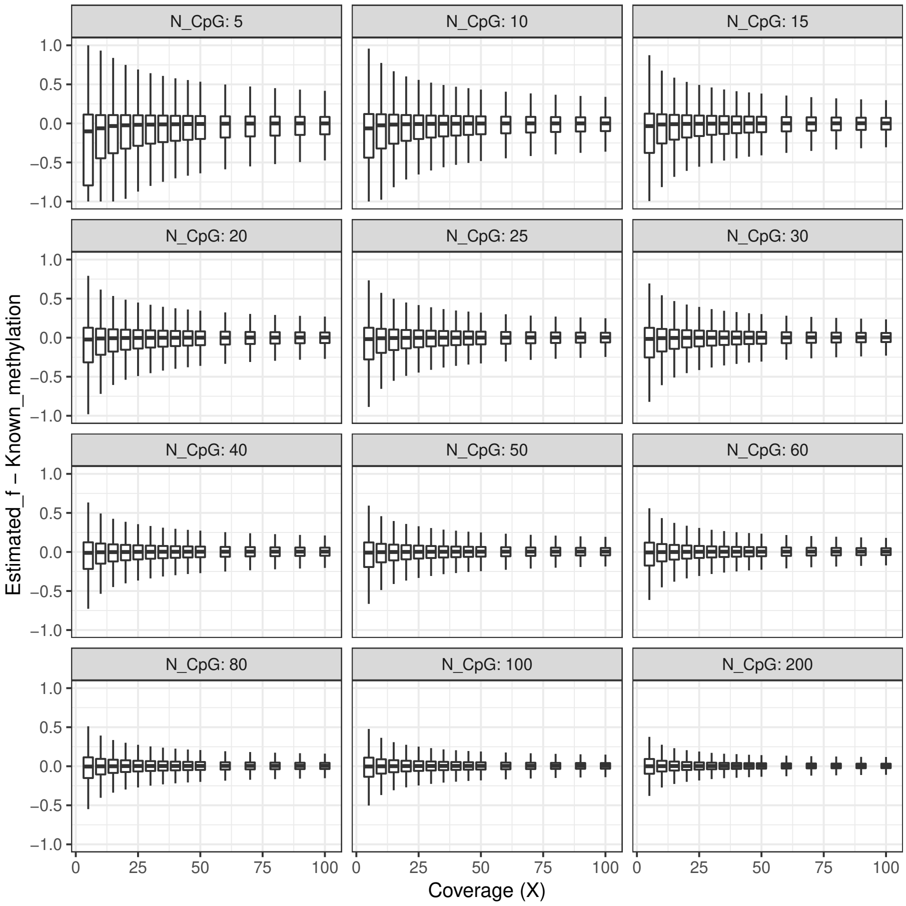

Figure 10: Boxplot of the accuracy of f (Estimated_f - known methylation) using the data from S-Ustishim

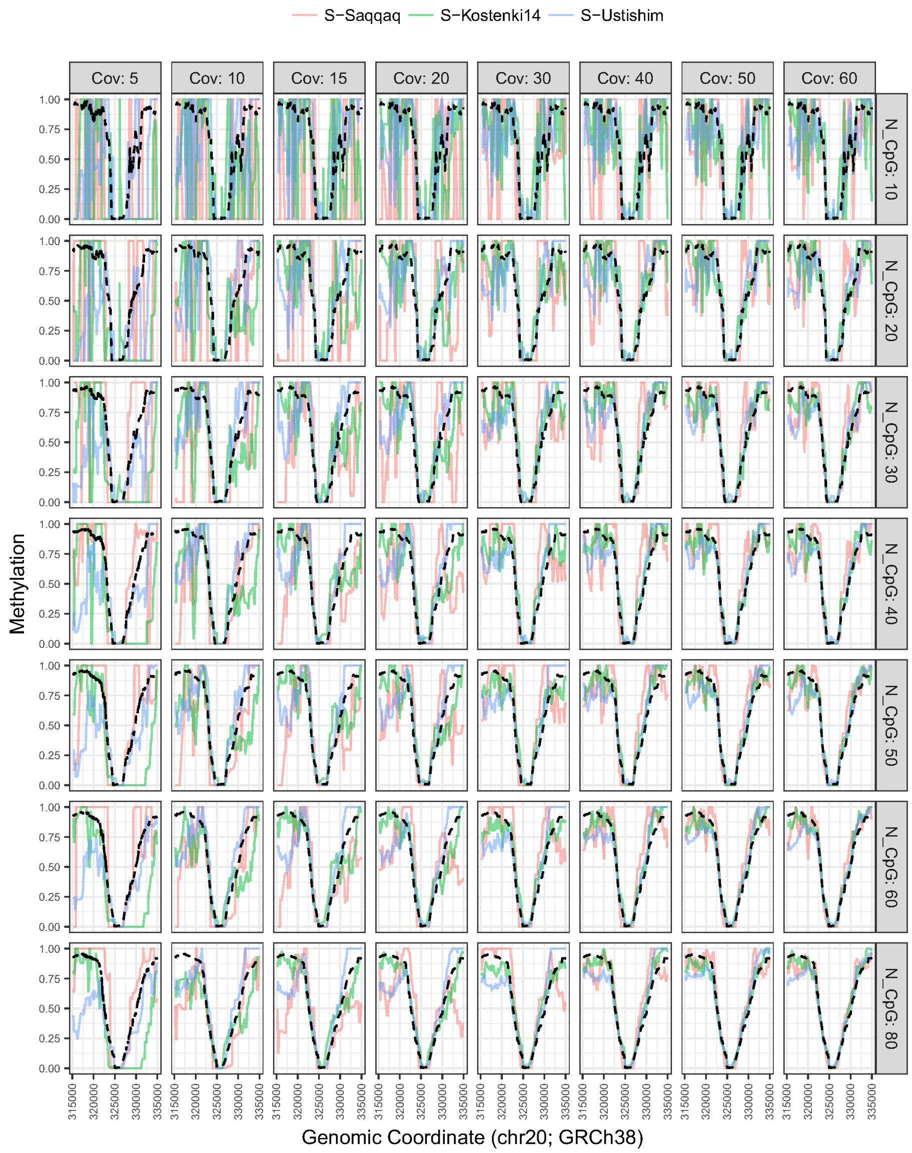

Figure 11: Estimates of f in a local genomic with a set of permutations of window size (N_CpG) and genomic coverage (coverage X). The expected methylation profile is shown as a black dashed line

2.1.5 Recovering methylation profiles with high densities of true variants

As described in equation 6, the model marginalizes over a set of seven dinucleotide genotypes (fig.1). We have implemented this, to reduce the noise from true variants on the estimate of f. In particular, the presence of a true $C \rightarrow T$ variant, in a CpG context, will increase the estimate of f dramatically, if the true

variants are observed as a series of deamination events. The same will happen with a $G \rightarrow A$ variant, as this will be confused with post mortem deamination events at the complementary CpG on the negative strand. To investigate if the expected regional methylation scores, f, can be recovered in our three simulated scenarios, despite the presence of true variants in CpG contexts, we have analyzed all CpG islands identified on chromosome 20 (GRCh38), with at least a single true variant in a CpG context in our simulated data. By providing DamMet with a prior probability of zero for observing any of the dinucleotide genotypes with an alternative allele ($G_{g \in (1,\dots,6}$), $C \rightarrow T$ observations can only be due to sequencing errors and deamination events. Alternatively, having a non-zero prior probability of observing an alternative allele, allows the model to discriminate true variants from deamination events and sequencing errors.

We find that having a non-zero prior improves the overall accuracy of f greatly (fig. 12; ALL).The increased accuracy is particularly pronounced for hypomethylated regions (fig. 12; Hypomethylation), whereas for hypermethylated regions, the effect is more moderate for S-Kostenki14 and S-Ustishim and even absent for S-Saqqaq. It is expected to see a less pronounced effect for hypermethylated regions, as the true variants will only increase the estimate of f towards already high estimated values, caped at one. We even find that using non-zero prior yields less accurate predictions at the minimal coverage (5X), due to the difficulty in disentangling true variants and post-mortem deamination, as both leaves similar signatures in the sequence data. However, with increasing coverage, a non-zero prior displays the minimal RMSD both in hypo- and hypermethylated contexts.

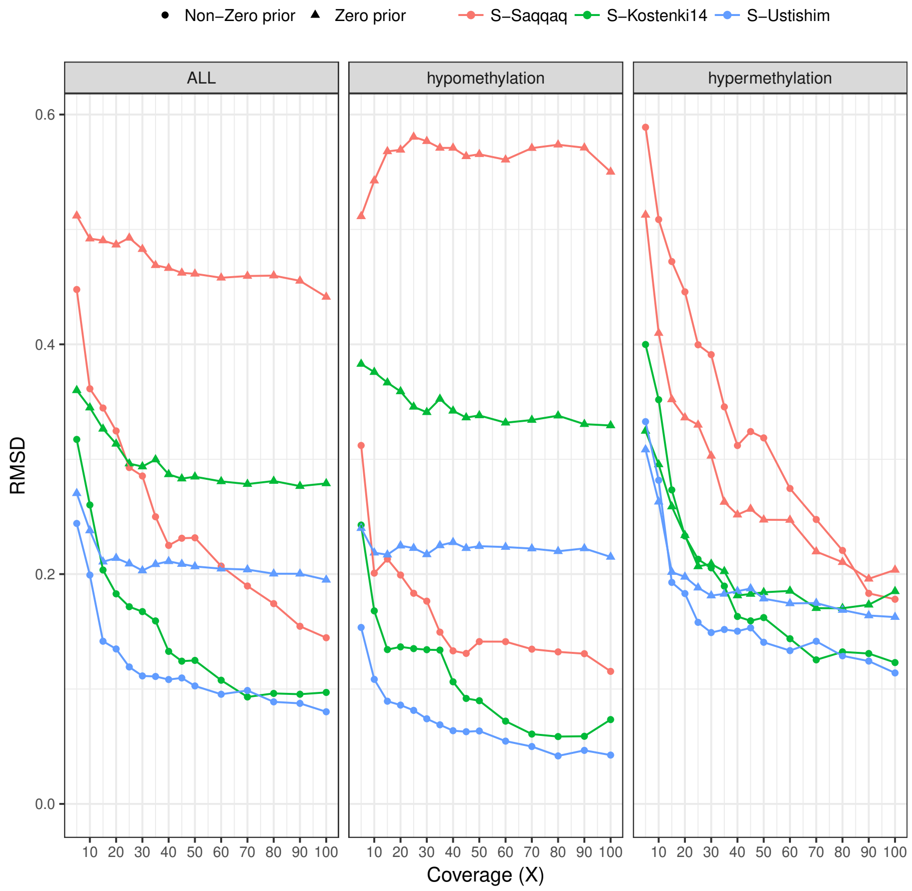

Figure 12: Root mean squared deviation (RMSD) of the accuracy of f against the known methylation level as a function of coverage (X) of all CpG islands with a least one SNP (ALL), those with less than 0.5 overall methylation levels (Hypomethylation), and lastly greater than 0.5 overall methylation (Hypermethylation).

2.2 DamMet applied to sequencing data underlying ancient specimens

We tested the accuracy of both steps of the model implemented in DamMet using simulated data and sequencing data from two ancient specimens, namely the 45,000 years old Ust'Ishim (hereafter referred to as Ustishim) sample sequenced to 42-fold coverage (Fu *et al.*, 2014) and the 50,000 years old Vi33 neanderthal sample sequenced to 30-fold coverage (Prüfer *et al.*, 2017). See Supplementary Results for an extensive description of the following analyses.

In this section, we apply DamMet to real published sequencing data underlying ancient specimens. We demonstrate that both our model steps of the model work with real sequencing data by applying them to Ustishim, a 42-fold coverage 45,000 years old anatomical modern human published by Fu and colleagues

(Fu *et al.*, 2014), and Vi33, a 30-fold coverage 50,000 years old Neanderthal published by Prüfer and colleagues (Prüfer *et al.*, 2017).

The first step of the algorithm in DamMet is to obtain position-specific deamination rates for each sample per read group. Next, we validate the estimate of f both chromosome-wide (chromosome 20) and locally. To validate the local estimates, we consider genomic regions with known methylation profiles such as CpG islands, their shores and shelves as well as promoter regions stratified by their %GC content and CpG density. However, we first briefly review one way of separating the majority of true variants from deamination events and why this is recommended prior to running DamMet.

2.2.1 Identifying true variants in ancient genomes

The statistical power to obtain an accurate estimate of f in DamMet, comes from the magnitude of the post-mortem deamination rates of methylated cytosines, and equally importantly from, from the difference in deamination rates between methylated and unmethylated cytosines. When true variants are incorporated, they increase the deamination rates of cytosines with a relatively larger contribution to unmethylated cytosines. This leads to a decrease in the absolute difference in deamination rates, which in turn results in a systematic underestimate of f regardless of the sequencing depth and/or window size. Applying filters to identify true variants is thus recommended prior to estimating the absolute deamination rates with DamMet.

To exclude variants in this study, we have used GATK HaplotypeCaller (version 4.0.8.1, (McKenna *et al.*, 2010)) and bcftools (version 1.8, (Li, 2011)) for filtering using the following commands,

```
FASTA=ref.fa
BAM=in.bam
outvcf=out.vcf
gatk HaplotypeCaller \
    --read-filter ReadLengthReadFilter \
    --min-base-quality-score 20 \
    --minimum-mapping-quality 30 \
    --TMP_DIR temp \
    --reference $FASTA \
    --input $BAM \
    -A FisherStrand \
    -A StrandBiasBySample \
    -A StrandOddsRatio \
    --output ${outvcf} \
    --interval-padding 0 \
    --create-output-variant-index false \
    --all-site-pls true \
    --genotyping-mode DISCOVERY \
    --min-read-length 25 \
    --max-read-length 2000

bcftools query -i 'QUAL>30 && INFO/DP>=5 &&
 (
```

```
    FORMAT/GT=="1/1" ||
    (REF=="C" && FORMAT/SB[*:3]>=2) ||
    (REF=="G" && FORMAT/SB[*:2]>=2)
)' -f "%CHROM %POS %REF %ALT\n" ${VCF} > ${VCF}.exclude
```

To designate a site as a true variant, besides having a minimum PHRED scale quality of 30 (1 out of 1000) and minimum depth of 5, one of the following criterion has to be met, (i) the site has to be homozygous for the alternative allele, (ii) observing two or more observations of the alternative allele on the negative strand with a cytosine residue as reference allele, (iii) observing two or more observations of the alternative allele on the positive strand with a guanine residue as reference allele. Sites that meets these criteria are excluded from any down stream analyses. We applied this filtering step to both Ustishim and Vi33.

2.2.2 Reconstruction of deamination rates using real data

In this section, we estimate the position-specific deamination rates of Ustishim and Vi33. First, we estimate the deamination rates of Ustishim where the data in each of the nine read groups has been generated following USER treatment (Briggs *et al.*, 2010) prior to building single stranded libraries (Meyer *et al.*, 2012). USER-treatment is a enzymatic process to excise all uracil residues present in the DNA molecule prior to library building and sequencing. Thus, with this treatment, we expected to see very low deamination rates for unmethylated cytosines. We also expected to see increased deamination rates of methylated cytosines towards not only the 5' but also the 3' of the DNA molecules due to the usage of a single stranded library protocol (Meyer *et al.*, 2012).

We find that the nine read groups follow largely the same trend, with the single exception of B5347 that shows an 50% increase in deamination rates at the first position 5' for methylated cytosines (fig 13). As expected the deamination rates of unmethylated cytosines are very low across all USER treated read groups.

In contrast to Ustishim, sequencing data underlying many published genomes arise from different library protocols, DNA extraction methods (e.g. (Rasmussen *et al.*, 2010)) and/or contain both USER-treated and nonUSER-treated sequencing data. Vi33 is an example of the latter, where eight of the nine read groups have not undergone any method to excise uracil residues prior to sequencing and the remaining read group (B8744) has been USER treated (Briggs *et al.*, 2010). In such case, we expect a larger inter group variance than found in Ustishim. Like Ustishim, all read groups found in Vi33 have been built with a single stranded library protocol (Meyer *et al.*, 2012).

First, we find high post-mortem deamination rates for both methylated and unmethylated cytosines for the eight untreated libraries, and low deamination rates solely for unmethylated cytosines in the USER treated read group (B8744). We also find that the treated read group have only been partly USER treated as illustrated by high deamination rates of unmethylated cytosines towards the termini of the DNA molecules (Rohland *et al.*, 2015). These results, found with DamMet, are all in line our the expectations for these read groups. Interestingly, we see an increased deamination of methylated cytosines in the 5' compared to unmethylated cytosines for the eight untreated read groups. This is likely due

the increased deamination rate of methylated cytosines (Seguin-Orlando *et al.*, 2015; Smith *et al.*, 2015).

Whether deamination rates should be estimated per read group or by merging all sequencing data into one is highly sample dependent. For samples such as Ustishim, the effect of stratifying by read group is probably marginal, however, for samples like Vi33 stratifying by read groups is crucial. If a sample consists of both USER treated and nonUSER treated read groups, by merging the sequencing data, the relative few deamination events originally observed in a USER treated read group, will not contribute proportionally to the estimate of f. In contrast, the deamination events found in nonUSER treated read groups would increase the estimate of f. This is so due to an incorrectly decreased probability of observing a deamination event for an unmethylated cytosine in the nonUSER treated read groups when merging.

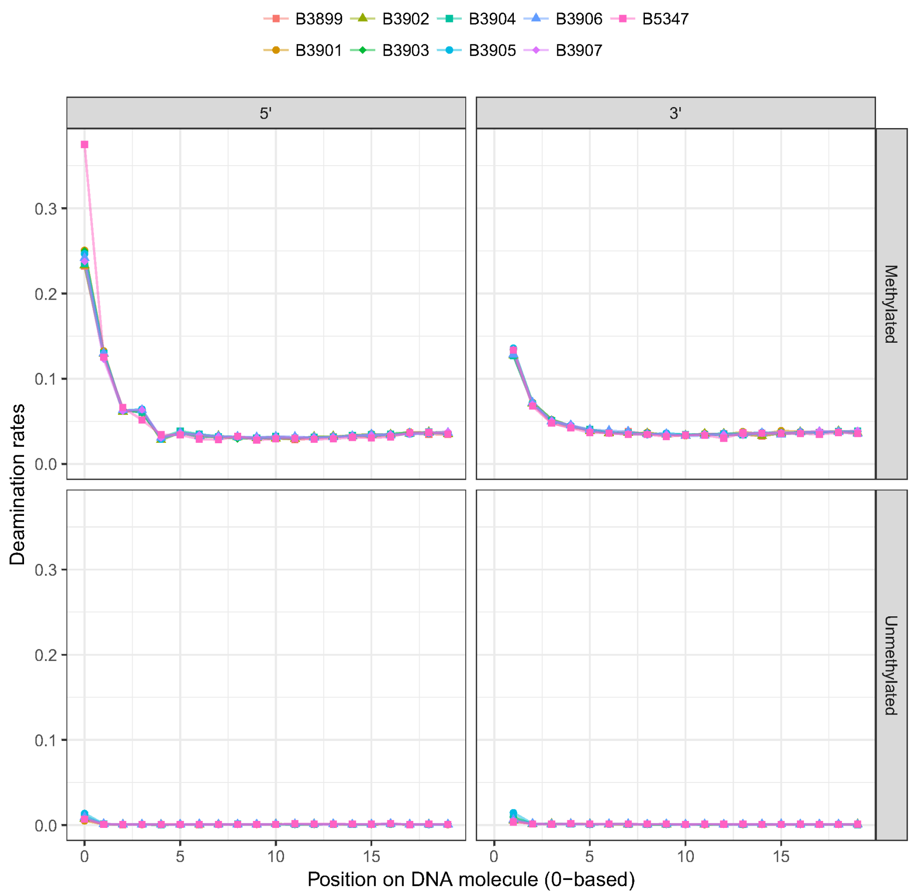

Figure 13: Estimated post-mortem deamination rates, from the sequencing data of Ustishim stratified by read groups, of (un)methylated cytosines for the first 20 positions of the 5' and 3' of a DNA molecule. Estimates are based on reads with a minimum length of 25bp

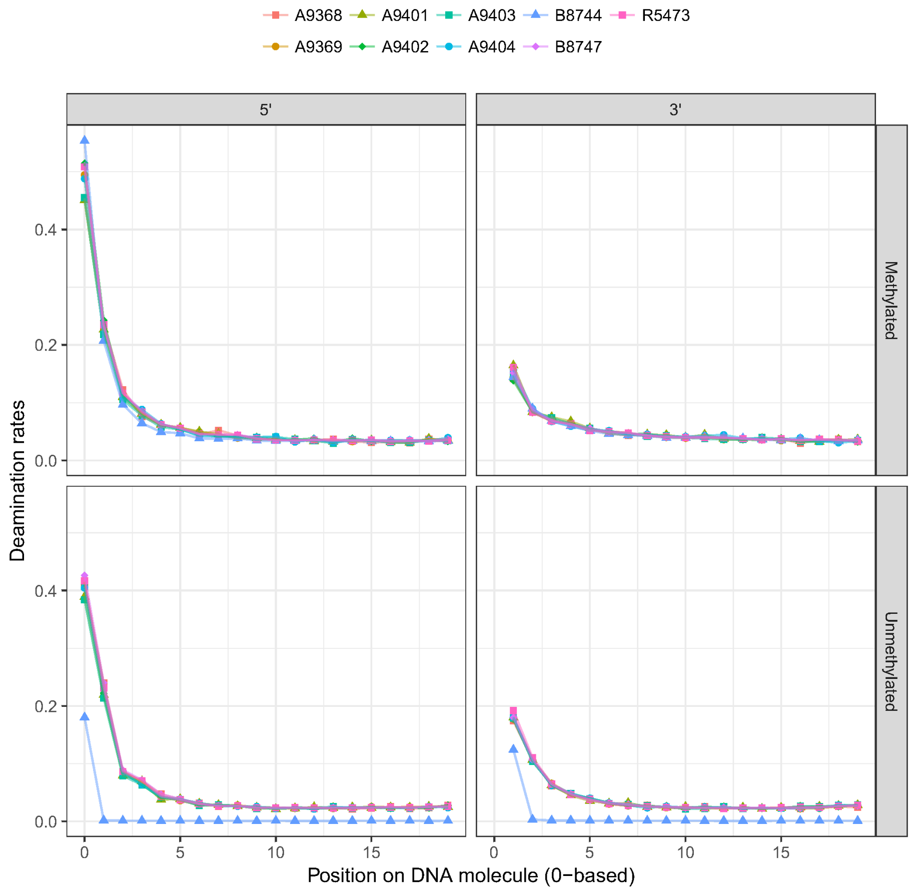

Figure 14: Estimated post-mortem deamination rates, from the sequencing data of the neanderthal sample Vi33 (Prüfer *et al.*, 2017) stratified by read groups, of (un)methylated cytosines for the first 20 positions of the 5' and 3' of a DNA molecule. Estimates are based on reads with a minimum length of 25bp

2.2.3 Chromosome-wide methylation levels

Using the position-specific post-mortem cytosines deamination rates estimated in section 2.2.2, we investigate if the expected chromosome-wide methylation levels can be recovered. In the absence of known true values for ancient specimens, we can expect a mean methylation level between 0.70 and 0.80 on the basis of available literature (Li and Zhang, 2014), and likely close to the modern WGBS methylome data used for simulations ENCFF103DNU from the ENCODE project with a mean methylation level of 0.748. Using all the sequencing data mapping to chromosome 20 (hg19) for these two samples, we find a mean value of 0.768 and 0.756 for Ustishim and Vi33, respectively. Both of these estimates are likely to overlap the actual chromosome-wide methylation level. Despite the majority of sequencing data in Vi33 originates from nonUSER treated libraries (8/9 read groups), DamMet still manages to find a chromosome wide

methylation level very close to the expectation. These results also confirm that the true variant filtering conducted in sec 2.2.1 removed a sufficient amount of noise to obtain accurate estimates of both methylated and unmethylated deamination rates.

2.2.4 Validation of f using genomic regions with known methylation profiles

In this section, we validate DamMet, using genomic regions with known methylation profiles from modern methylation data. These include the hypomethylated (i) CpG islands, followed by shores (2kb), then shelves (2kb) where we expect a bell shaped methylation profile with gradual increment of methylation as the distance from the hypomethylated CpG island increases. We also analyzed genomic (ii) promoter regions, defined as 1kb downstream and 400bp upstream of the transcriptional start site, stratified by their %GC and CpG density into three categories (HIGH, INTERMEDIATE, and LOW). Here, an inverse correlation between the promoter categories and their methylation levels is expected (Ball *et al.*, 2009). We have investigated these genomic regions for both Ustishim and Vi33 and for comparison, the WGBS methylome data used for the simulations is also included (see sec 2.1.1;ENCFF103DNU). The modern data is merely provided as a comparative baseline for the two ancient samples, as a number of different factors, notwithstandingly the huge temporal difference, can have modified some of the genomic regions investigated.

We find that the trend in the two ancient samples follow accurately that of the modern WGBS data (fig 15). However, a slight, but consistent, underestimate of f is observed in regions with a low/intermediate CpG density. This is a result of decreased statistical power for estimating f when few CpG data points are available per genomic window. This bias can be mitigated by increasing the window size and/or sequencing effort. Nonetheless, the RMSD is still quite low (Ustishim: 0.058; Vi33: 0.10) for our ancient samples. As expected, Vi33 show the highest RMSD as the majority of the sequencing data is nonUSER treated (8/9 read groups). For a nonUSER treated DNA molecule, a deamination event, or the absence thereof, provides extremely reduced statistical power to allow an accurate estimating of f compared to USER-treated DNA molecule. The drop in power is a consequence of including unmethylated cytosines that deaminated to uracil residues and sequenced as thymine residues.

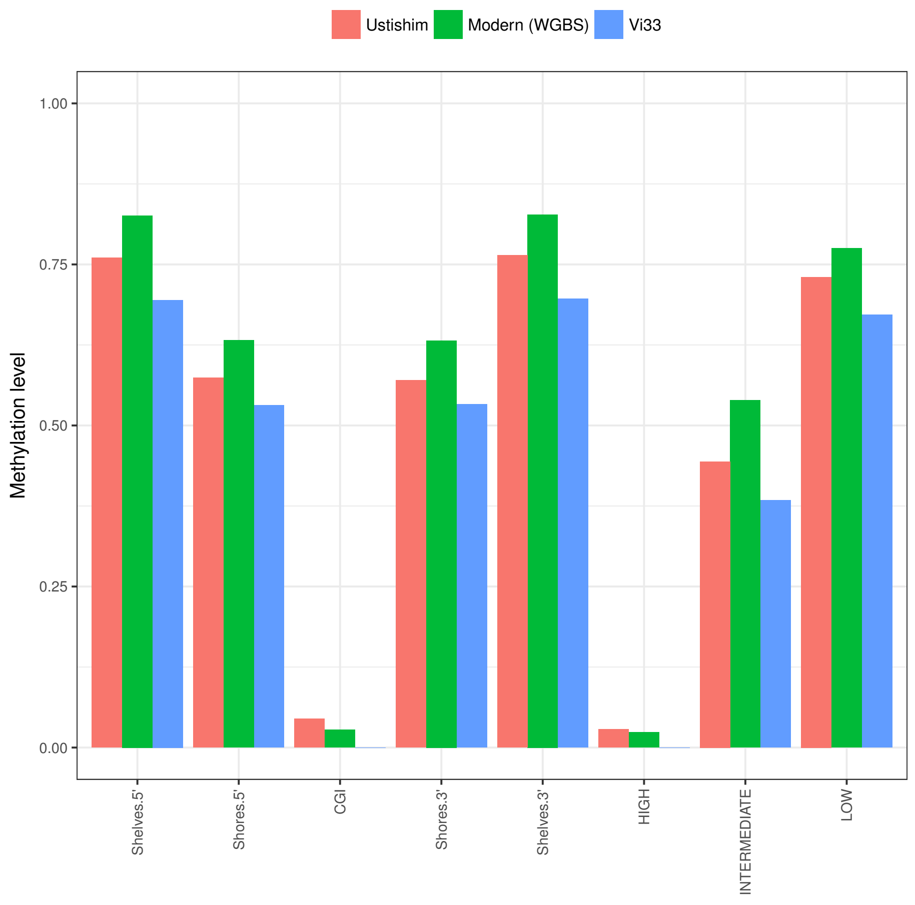

Figure 15: Genomic regions with known stable methylation levels including CpG islands, their shores and shefles, and promoter regions stratified by their %GC content and CpG density (HIGH, INTERMEDIATE, and LOW). Modern (green) is provided as a comparative baseline on the basis of modern methylation data.

References

Ball, M. P., Li, J. B., Gao, Y., Lee, J.-H., LeProust, E. M., Park, I.-H., Xie, B., Daley, G. Q., and Church, G. M. (2009). Targeted and genome-scale strategies reveal gene-body methylation signatures in human cells. *Nature biotechnology*, **27**(4), 361–368.

Briggs, A. W., Stenzel, U., Johnson, P. L. F., Green, R. E., Kelso, J., Prüfer, K., Meyer, M., Krause, J., Ronan, M. T., Lachmann, M., and Pääbo, S. (2007). Patterns of damage in genomic DNA sequences from a Neandertal. *Proceedings of the National Academy of Sciences of the United States of America*, **104**(37), 14616–14621.

Briggs, A. W., Stenzel, U., Meyer, M., Krause, J., Kircher, M., and Pääbo, S. (2010). Removal of deaminated cytosines and detection of in vivo methylation in ancient DNA. *Nucleic acids research*, **38**(6), e87.

Fu, Q., Li, H., Moorjani, P., Jay, F., Slepchenko, S. M., Bondarev, A. A., Johnson, P. L. F., Aximu-Petri, A., Prüfer, K., de Filippo, C., Meyer, M., Zwyns, N., Salazar-García, D. C., Kuzmin, Y. V., Keates, S. G., Kosintsev, P. A., Razhev, D. I., Richards, M. P., Peristov, N. V., Lachmann, M., Douka, K., Higham, T. F. G., Slatkin, M., Hublin, J.-J., Reich, D., Kelso, J., Viola, T. B., and Pääbo, S. (2014). Genome sequence of a 45,000-year-old modern human from western Siberia. *Nature*, **514**(7523), 445–449.

Gokhman, D., Lavi, E., Prüfer, K., Fraga, M. F., Riancho, J. A., Kelso, J., Pääbo, S., Meshorer, E., and Carmel, L. (2014). Reconstructing the DNA methylation maps of the Neandertal and the Denisovan. *Science*, **344**(6183), 523–527.

Hanghøj, K., Seguin-Orlando, A., Schubert, M., Madsen, T., Pedersen, J. S., Willerslev, E., and Orlando, L. (2016). Fast, accurate and automatic ancient nucleosome and methylation maps with epipaleomix. *Molecular biology and evolution*, **33**(12), 3284–3298.

Huang, W., Li, L., Myers, J. R., and Marth, G. T. (2012). ART: a next-generation sequencing read simulator. *Bioinformatics*, **28**(4), 593–594.

Li, E. and Zhang, Y. (2014). DNA Methylation in Mammals. *Cold Spring Harbor Perspectives in Biology*, **6**(5).

Li, H. (2011). A statistical framework for SNP calling, mutation discovery, association mapping and population genetical parameter estimation from sequencing data. *Bioinformatics*, **27**(21), 2987–2993.

Li, H. and Durbin, R. (2009). Fast and accurate short read alignment with Burrows–Wheeler transform. *Bioinformatics*, **25**(14), 1754–1760.

Lindahl, T. (1993). Instability and decay of the primary structure of DNA. *Nature*, **362**(6422), 709–715.

McKenna, A., Hanna, M., Banks, E., Sivachenko, A., Cibulskis, K., Kernytsky, A., Garimella, K., Altshuler, D., Gabriel, S., Daly, M., and DePristo, M. A. (2010). The Genome Analysis Toolkit: a MapReduce framework for analyzing next-generation DNA sequencing data. *Genome research*, **20**(9), 1297–1303.

Meyer, M., Kircher, M., Gansauge, M.-T., Li, H., Racimo, F., Mallick, S., Schraiber, J. G., Jay, F., Prüfer, K., de Filippo, C., Sudmant, P. H., Alkan, C., Fu, Q., Do, R., Rohland, N., Tandon, A., Siebauer, M., Green, R. E., Bryc, K., Briggs, A. W., Stenzel, U., Dabney, J., Shendure, J., Kitzman, J., Hammer, M. F., Shunkov, M. V., Derevianko, A. P., Patterson, N., Andrés, A. M., Eichler, E. E., Slatkin, M., Reich, D., Kelso, J., and Pääbo, S. (2012). A high-coverage genome sequence from an archaic Denisovan individual. *Science*, **338**(6104), 222–226.

Pedersen, J. S., Valen, E., Velazquez, A. M. V., Parker, B. J., Rasmussen, M., Lindgreen, S., Lilje, B., Tobin, D. J., Kelly, T. K., Vang, S., Andersson, R., Jones, P. A., Hoover, C. A., Tikhonov, A., Prokhortchouk, E., Rubin, E. M., Sandelin, A., Gilbert, M. T. P., Krogh, A., Willerslev, E., and Orlando, L. (2014). Genome-wide nucleosome map and cytosine methylation levels of an ancient human genome. *Genome research*, **24**(3), 454–466.

Prüfer, K., de Filippo, C., Grote, S., Mafessoni, F., Korlević, P., Hajdinjak, M., Vernot, B., Skov, L., Hsieh, P., Peyrégne, S., *et al.* (2017). A high-coverage neandertal genome from vindija cave in croatia. *Science*, **358**(6363), 655–658.

Rasmussen, M., Li, Y., Lindgreen, S., Pedersen, J. S., Albrechtsen, A., Moltke, I., Metspalu, M., Metspalu, E., Kivisild, T., Gupta, R., Bertalan, M., Nielsen, K., Gilbert, M. T. P., Wang, Y., Raghavan, M., Campos, P. F., Kamp, H. M., Wilson, A. S., Gledhill, A., Tridico, S., Bunce, M., Lorenzen, E. D., Binladen, J., Guo, X., Zhao, J., Zhang, X., Zhang, H., Li, Z., Chen, M., Orlando, L., Kristiansen, K., Bak, M., Tommerup, N., Bendixen, C., Pierre, T. L., Grønnow, B., Meldgaard, M., Andreasen, C., Fedorova, S. A., Osipova, L. P., Higham, T. F. G., Ramsey, C. B., Hansen, T. V. O., Nielsen, F. C., Crawford, M. H., Brunak, S., Sicheritz-Pontén, T., Villems, R., Nielsen, R., Krogh, A., Wang, J., and Willerslev, E. (2010). Ancient human genome sequence of an extinct Palaeo-Eskimo. *Nature*, **463**(7282), 757–762.

Renaud, G., Hanghøj, K., Willerslev, E., and Orlando, L. (2017). gargammel: a sequence simulator for ancient DNA. *Bioinformatics*, **33**(4), 577–579.

Rohland, N., Harney, E., Mallick, S., Nordenfelt, S., and Reich, D. (2015). Partial uracil-DNA-glycosylase treatment for screening of ancient DNA. *Philosophical transactions of the Royal Society of London. Series B, Biological sciences*, **370**(1660), 20130624.

Seguin-Orlando, A., Korneliussen, T. S., Sikora, M., Malaspinas, A.-S., Manica, A., Moltke, I., Albrechtsen, A., Ko, A., Margaryan, A., Moiseyev, V., Goebel, T., Westaway, M., Lambert, D., Khartanovich, V., Wall, J. D., Nigst, P. R., Foley, R. A., Lahr, M. M., Nielsen, R., Orlando, L., and Willerslev, E. (2014). Paleogenomics. Genomic structure in Europeans dating back at least 36,200 years. *Science*, **346**(6213), 1113–1118.

Seguin-Orlando, A., Gamba, C., Der Sarkissian, C., Ermini, L., Louvel, G., Boulygina, E., Sokolov, A., Nedoluzhko, A., Lorenzen, E. D., Lopez, P., McDonald, H. G., Scott, E., Tikhonov, A., Stafford, Jr, T. W., Alfarhan, A. H., Alquraishi, S. A., Al-Rasheid, K. A. S., Shapiro, B., Willerslev, E., Prokhortchouk, E., and Orlando, L. (2015). Pros and cons of methylation-based enrichment methods for ancient DNA. *Scientific reports*, **5**, 11826.

Smith, R. W. A., Monroe, C., and Bolnick, D. A. (2015). Detection of Cytosine methylation in ancient DNA from five native american populations using bisulfite sequencing. *PloS one*, **10**(5), e0125344.

Svanberg, K. (2002). A class of globally convergent optimization methods based on conservative convex separable approximations. *SIAM Journal on Optimization*, pages 555–573.
