# Supplementary material for: DamMet: ancient methylome mapping accounting for errors, true variants, and post-mortem DNA damage
Source: Gigascience. 2019 Apr 20;8(4):giz025. doi: 10.1093/gigascience/giz025 (PMC6474913; doi:10.1093/gigascience/giz025)
Supplement: Response_to_Reviewer_Comments_Original_Submission.pdf [file giz025_response_to_reviewer_comments_original_submission.pdf]

Author's Response To Reviewer Comments

Close

Dear Editor,

We were pleased to receive such positive feedback on our manuscript entitled “DamMet, a full probabilistic model for mapping ancient methylomes”. We would like to thank you and both reviewers for their valuable feedback, which contributed improve and clarify our revised manuscript. In short:

• We have fixed all noted typos and latex issues in the manuscript, and scrutinized the text to correct pending grammar and spelling issues.

• We have emphasized throughout the whole manuscript that DamMet estimates methylation levels in CpG context only. Addressing other sequence-dependent methylation contexts would require further developments that are beyond the scope of the present study (eg plant DNA methylation can occur in specific tri-nucleotide sequence contexts, thus implying extensive changes in our current model, which can only accommodate dinucleotide states). We also note that the vast majority of ancient genomes generated so far consist of human individuals (and to a lower extent of domestic mammals, such as dogs, horses and goats). The model presented here will thus have direct applicability to available data.

• We have made a tag release of DamMet, added a pull request to bioconda, and made the simulated data used in this study publicly available. Furthermore, a link to the ftp server where the BAM files of Ust-ishim and Vi33 are located have been included in the manuscript. Finally, we have registered the software on SciCrunch.org and the RRID (SCR_016959) is now included in the manuscript.

We hope that you will find our revised manuscript suitable for publication in GigaSciences. Please do not hesitate to contact us, should anything remain unclear.

Reviewer #1: Dear authors,

thank you very much for making the method available and taking the time to perform such extensive research into this topic! I broadly separated my comments and questions in a set of questions + typos. I furthermore have some questions regarding the supplementary material, that I'd like to be included here as well.

POINT 1.
Typos (please find these highlighted in the PDF as well, let me know if these can't be opened by you).

- p4, l 57, method' accuracy
- p5, l19 "deaminatino"
- p7, l10 missing "s"
- p7, l51 a "to" too much
- p7, l4 "analyze" => analyzes
-p7, l5 , "it can be parallelized easily per chromosome"

ANSWER 1.
>> We have now corrected all such typos (as well as others).

POINT 2.
Questions to authors:
Q1.) I already used and applied gargammel to simulate ancient DNA sequences for some time and found it to be quite reliable. You mentioned that you had to add several adjustments to simulate methylation specific PMD patterns in your case. Can you demonstrate and/or explain a bit more in detail what makes you confident that you don't develop "egg and hen" to be compatible here? In other words: Gargammel to produce output based on a synthetic model that is then interpreted/analyzed well using DeamMet?

ANSWER 2.

>> First, we should stress that we did not make adjustments to the core pipeline of gargammel, which remains identical to the version originally released (Renaud et al. 2016). Instead, we have added another feature so as to simulate post-mortem cytosine deamination at both methylated and un-methylated cytosines. Such a feature was not present in the version of gargammel originally published, which prevented using the read simulator for testing eg the sensitivity of any method to detect ancient DNA methylation marks and how their presence could affect any type of downstream data analyses. Our new procedure is fully available in a new release of the software. In short, it can take two post-mortem deamination profiles as input: one for the occurence of post-mortem deamination events at methylated cytosines and one at unmethylated cytosines. These provide position-specific post-mortem deamination probabilities in both contexts and help apply the correct underlying post-mortem degradation kinetics depending on the context considered. The only other feature now added pertains to the possibility of using multiple genomes as input so as to simulate DNA coming from multiple cells. Indeed, extensive evidence shows that only a fraction of the cells in any given tissue show similar methylation profile at any given genomic position.

More specifically, we consider that post-mortem deamination events are stochastic. The frequency of these events depends on the DNA sequence context (methylated CpG deaminate faster than other CpN dinucleotide contexts) and the location along the DNA molecule (eg overhanging ends are more likely to be deaminated than double-stranded regions of the ancient DNA molecule). Generating synthetic datasets with gargammel proceeds by first adding post-mortem deamination events to ancient DNA fragments using a Bernoulli trial where the probability of deamination in a given context is based on deamination frequencies provided by the user. This procedure resembles the stochastic process of post-mortem deamination events affecting cytosine residues.

Although several unknown artifacts might not be accounted for by our methodology yet, we provide evidence that the simulations are realistic. First, we show that our estimates of f, the regional values of DNA methylation in a given genomic window, show low precision and accuracy at low sequencing depth. This correlation is expected as the probability of observing a post-mortem deamination event at a given site increases with sequencing depth. The sensitivity to observe the end product of post-mortem cytosine deamination at limited sequencing depths is therefore extremely reduced, hence, our estimates become extremely imprecise. Reciprocally, we show that DamMet performs well on high coverage synthetic data but also produces results on par with expectations when applied to real data (ie the two ancient samples analyzed, Ust-ishim, 45X and Vi33, 30X). We thus conclude that the methodology present is valid based on both synthetic simulations and real data. Taken together, the damage patterns present in the synthetic data are likely to reflect the damage patterns present in sequence data underlying ancient specimens, and DamMet is unlikely to show good performance on data overfitting the expectations of its underlying statistical model.

As a more general note, there are currently no wet-lab procedure that can generate genome-wide empirical DNA methylation data in an ancient individual in an unbiased and cost-effective manner. Amongst the two methods hitherto tested, MBD enrichment (Seguin-Orlando et al. 2015, Smith et al. 2015) show strong limitations pertaining to the length of the DNA molecules preserved, while bisulfite DNA sequencing (Llamas et al. 2012, Smith et al. 2015) requires DNA amounts not compatible with post-mortem DNA decay. Therefore, any methodology aimed at validating statistical methods for detecting ancient DNA methylation marks MUST rely on indirect evidence, hence, simulated synthetic datasets and real data at sufficient coverage so as to obtain accurate post-mortem deamination rates in relevant sequence contexts.

POINT 3.
Q2.) Could you please provide some more details on the sample data you used? Referring to the publication is ok, but as data is e.g. uploaded in BAM/FASTQ format in some cases, I'd be happy to see the ENA/NCBI/SRA/ (whichever platform these were uploaded) in the manuscript to make the procedure more reproducible.

ANSWER 3.
>> In addition to referring to the corresponding individual publications, we have now added links to the ftp servers where the BAM files can be directly retrieved. This provides users with direct access to the underlying data.

"... the 45k year-old Ust'Ishim (42-fold coverage; http://cdna.eva.mpg.de/ust-ishim/BAM/)"
and
"... the 50k year-old Vi33 Neanderthal (30-fold coverage;

http://cdna.eva.mpg.de/neandertal/Vindija/bam/)”

POINT 4.
Q3.) Did you by any chance have a look at how much influence in methylation retrieval can be seen when data is processed to BAM level using different methods? I suppose you used the BAM files as provided by the respecitve authors, but did you have a look at this in general?

ANSWER 4.
>> The reviewer is correct in assuming that we have used the BAM files provided from the respective authors with no further modification. We have not explored how different filtering methods would affect our predictions, given the almost unlimited range of possibilities to consider, which would go well beyond the scope of this study. We also note that the two BAM files considered are filtered to the highest possible quality (and indeed show error rates on par with what observed on modern DNA data). Considering BAM files of more limited quality would likely reduce the performance of DamMet.

POINT 5.
Q4.) Can you please provide runtimes for an entire sample? You only mentioned runtimes for chromosome 1 of one sample, but I miss memory/resource requirements for an entire sample, which could give users a hint on which kind of system they might need to use for an analysis in general. As you probably did an entire benchmark/runtime for internal usage (?) anyways, this shouldn't be a big mess for you to add.

ANSWER 5.
>> The runtime provided now reflects the time to generate the entire methylome for the two ancient samples analyzed in this study using a single CPU. We have also added memory usage information.

“The reconstruction of the entire methylome of Ust-Ishim and Vi33 took 11h and 8h on a single CPU (E5-2683 v4 @ 2.10GHz) with memory usages peaking at 10GB and 9GB, respectively.”

POINT 6.
Q5.) Additional ideas/comments:
As this is a computational method, I was trying to compile and run the method on a small sample (LBK-Stuttgart, just chromosome 22) and was happy to see that this resulted in interpretable results in general. As more and more researchers would probably want to use your method in the near future, I'd be happy to see the following as well:
- a stable release on GitLab (no need for Github, but a fixed release https://docs.gitlab.com/ee/workflow/releases.html)
- a bioconda recipe for your package once you have that stable release. This eases the pain of installation for many (!) users substantially, also giving you much more credit to have a nicely packaged and reproducibly usable method out there. As the requirements for your tool are already there in bioconda, you'd only have to write a small build + meta.yaml script to get this done. https://bioconda.github.io/contribute-a-recipe.html

I opened issues for these steps in your repository to let you know...

ANSWER 6.
>> Thanks for these suggestions and for adding them to the repository. We made a stable version of DamMet (tag:1.0.1) available. We have also added a pull request to add DamMet to the bioconda-recipes.

POINT 7.
Q6.) There are some latex issues in the supplementary material. Maybe try reducing the size of some of the formulas to fit into a single page width (or get these on a separate page in widescreen).

ANSWER 7.
>> We have fixed the latex issues by splitting the equations into multiple lines.

POINT 8.
Q7.) In 2.1.2 You specify "All simulated BAM can be downloaded from XXXXX." - could you make these available? (also typo, "files" missing)

ANSWER 8.

>> We have made the BAM files with synthetic data available at https://sid.erda.dk/public/archives/29e740f2715b6353deea88989723b950/published-archive.html and added 'files' to the sentence. It now reads:

"All simulated BAM files can be downloaded from ERDA, an electronic scientific data repository provided and maintained by the University of Copenhagen (LINK)."

POINT 9.
Reviewer #2: Dear authors,
Q9.) The title of this paper describes a new method (and its implementation) for mapping ancient methylomes. However, I think it rather describes a model for measuring post-mortem deamination rates at methylated and unmethylated sites (and this is described in the abstract and through all article). The symmetric methylome regions can be derived by comparison with cross-samples. Therefore, in my opinion, the title might be a little confusing.

ANSWER 9.
>> The method underlying DamMet follows a two step procedure. First, we obtain a maximum likelihood estimate of post-mortem deamination rates at both methylated and unmethylated sites, given that these site categories show different post-mortem deamination rates. The second step makes use of these rates to obtain a maximum likelihood estimate of methylation in CpG context. The methylation estimates are thus not obtained by cross-sample comparison, instead they are obtain per individual. Cross-sample comparisons would reveal eg differentially methylated regions but such identifications will always come downstream of DamMet. Our title aimed at emphasizing the main difference with the approach developed in DamMet and previous methodology. DamMet indeed is the only and unique procedure accounting for sequencing errors, mapping errors and differential post-mortem Cytosine deamination rates at methylated and unmethylated sites in an explicit statistical model. All previous approaches, including the one that we previously released in epiPALEOMIX (Hanghøj et al. 2016), calculated DNA methylation scores on the basis of naive counts at positions a priori considered to derive from post-mortem deamination. While providing full focus on the statistical model, our previous model however missed the ultimate end product of DamMet, namely that DNA methylation maps are inferred. We thus have now edited our original title to also reflect this:

DamMet: ancient methylome mapping accounting for errors, true variants and post-mortem DNA damage

POINT 10.
Q10.) Another point is the absence of MapDamage in the article (at least the version 2), namely Jónsson, Hákon, et al. "mapDamage2. 0: fast approximate Bayesian estimates of ancient DNA damage parameters." Bioinformatics 29.13 (2013): 1682-1684.
What is new regarding MapDamage2? What was used from MapDamage2? One paragraph to explain to the community the foundations would be kind, even if major changes have been applied and other sub-methods added.

ANSWER 10.
>> The method implemented in MapDamage2 makes use of mismatch counts when comparing DNA sequencing data to a predefined target reference genome. The underlying statistical model provides four key parameters of post-mortem DNA decay, namely the length of the overhang, nick frequency, and cytosine deaminations rates in both double-stranded and overhanging DNA contexts. None of these parameters are estimated nor used in DamMet. Instead, we identify position-specific deamination profiles at methylation and unmethylated cytosines through the full likelihood model described in-depth in supplementary section 1.2. This model is NOT the one we originally described while releasing mapDamage2. As a matter of fact, the latter was published in 2013, ie one year before we realized that ancient DNA methylation marks could be predicted leveraging the CpG>TpG mis-incorporation present in ancient DNA datasets. The model underlying mapDamage2 does NOT factor in the possibility of differential cytosine deamination kinetics at both methylated and unmethylated sites.

It is noteworthy that DamMet is not meant to replace MapDamage2. Whereas MapDamage2 estimates key parameters of DNA degradation patterns, DamMet makes use of the degradations patterns to reconstruct ancient methylomes in CpG context. It also is becoming increasingly common to treat ancient DNA extracts with enzymes such as USER to remove uracil residues from the sequencing data

prior to library preparation. This increases the accuracy of the data generated by reducing the impact of nucleotide mis-incorporations pertaining to post-mortem DNA decay. Following this treatment, some of the underlying assumptions of the mapDamage2 model (eg the average overhang length follows a geometric distribution) can be violated. The underlying data thus becomes inappropriate for mapDamage2 but optimal for DamMet as we show that USER treatment improves ancient methylome mapping. Reciprocally, in the absence of USER treatment, DamMet becomes largely un-applicable unless high coverage is achieved. In this case, mapDamage2 remains useful to quantify important parameters of post-mortem DNA damage.

POINT 11.
Q11.) In theory, the words "ancient methylomes" are referred to both symmetric and asymmetric methylation. The method seems to detect only symmetric methylations (CpG), while I do not see any reference regarding asymmetric methylation (CpNpNp), namely what occurs in plants. There are also characteristics of particular methylations, namely from invertebrates and plants. See, for example, Zemach, Assaf, et al. "Genome-wide evolutionary analysis of eukaryotic DNA methylation." Science 328.5980 (2010): 916-919. Therefore, please clarify if the method only works for the symmetric type.

ANSWER 11.
>> The reviewer is correct, DamMet only detects methylation in CpG context. For mammals, cytosines in CpG contexts carry the vast majority of methyl-groups, we therefore focused on this specific context. We are aware that asymmetric methylation is also present, in particular in plants. An extension to DamMet to estimate asymmetric methylation is possible and we are open for developing it should the need arise, however, CpG methylation was the main focus of this study and we have clarified that in the manuscript. See also the last paragraph of ANSWER 2 pertaining to the availability of ancient genome data, which is hitherto almost entirely limited to humans and animal domesticates, where symmetric methylation dominates.

Below are highlights from the manuscript where we emphasize that DamMet produces estimates of DNA methylation in CpG context:

Abstract:
“Here, we present DamMet, an open-source software retrieving maximum likelihood estimates of regional CpG methylation levels from ancient DNA sequencing data.”

Introduction:
“Here, we present DamMet, a software returning regional maximum likelihood estimates (MLE) of CpG methylation from high-throughput DNA sequencing data obtained from an ancient specimen.”

Methods:
“The second step makes use of D, obtained in the first step, to recover a MLE of f, the fraction of methylated CpGs in a given genomic window.”

Methods (Simulation):
“The methodology consists first of simulating sequencing data from 100 diploid genomes, where each CpG position is flagged as methylated or unmethylated.”

Results:
“The likelihood function is maximized to obtain f, includes all dinucleotide read observations covering CpGs in a given genomic window.”

“The likelihood function is maximized to obtain f, includes all dinucleotide read observations covering CpGs in a given genomic window.”

“Lastly, we demonstrated that DamMet obtains accurate methylation estimates in regions with a high density of true variants located in CpG contexts by incorporating the possibility of observing true dinucleotide variants in the likelihood function (Supplementary Results 2.1.5)”

Finally, we also acknowledge asymmetric methylation marks as possible development perspectives in the future:
Given that the vast majority of high-coverage ancient genomes are from human and domesticated animal specimens, the current implementation of DamMet estimates methylation in symmetric CpG contexts. This is by far the most dominant context associated with methylation in mammals.

Nonetheless, following an increase in high-coverage ancient genomes, for which methylation occurs predominantly in asymmetric sequence contexts (e.g. CpNpN that often occurs in plants [18]), MLE estimates of f in such contexts could be added in future releases of DamMet.

POINT 12.
Q12.) In the article and Supplementary Material, I've seen that the method underestimates certain values. I've no problems with this, namely because underestimating means that when the tool reports any signal it means that it exists. Therefore, no false negatives are identified, which is better than having false positives. An example of non-overestimation is present in, for example, Pratas, Diogo, et al. "Metagenomic Composition Analysis of an Ancient Sequenced Polar Bear Jawbone from Svalbard." Genes 9.9 (2018): 445.
Still, in the underestimation, the default mapping values (bwa) have been used (only seed disabled), which for ancient DNA, might not be appropriate. Increasing the edit distance and using relaxed thresholds might increase the mapping rate, especially in reads with length below 40. Please, see Schubert, Mikkel, et al. "Improving ancient DNA read mapping against modern reference genomes." BMC Genomics 13.1 (2012): 178.
So, I'm wondering why appropriate parameters for aDNA were not used in this article? Does this process increase the ambiguity while using DamMet? Please, be clear here.

ANSWER 12.
>> The reviewer is correct that mapping parameters should be carefully considered while analyzing ancient DNA data. We are fully aware of the best standard, especially as the article referred to (Schubert et al. 2012) was developed by some of us. In this particular article, we demonstrated that disabling seeding in BWA was optimal for ancient DNA data. This is so because post-mortem DNA damage tend to take place within overhanging ends of ancient DNA molecules, causing inflated sequencing errors towards read termini. Seeding would introduce too strict edit distance thresholds while attempting to match the seed subword against the reference genome, thus, reducing sensitivity and inflating false negatives. The mapping parameters used in this study are on par with best procedures and relevant literature, including our own (eg see PALEOMIX, Schubert et al. 2014) but not restricted to our own (eg Botigue et al. 2018, Moreno-Mayar et al. 2018, Wright et al. 2018). As a general note, the problem of defining optimal and versatile mapping parameters for ancient DNA data is a difficult one. Increasing the allowed edit distance will definitely reduce the slight underestimate of deamination rates of methylated cytosine residues. However, by increasing the allowed edit distance, the fraction of exogenous DNA molecules from microbes mapping to the target reference and spuriously mapping reads will inevitably increase. This was demonstrated by some of us (Renaud et al. 2016) as well as others (eg Taron et al. 2018; https://www.ncbi.nlm.nih.gov/pmc/articles/PMC5867878/; figure 2).

POINT 13.
Q13.) There isn't a direct comparison with other methods, namely from Gokhman, David, et al. "Reconstructing the DNA methylation maps of the Neanderthal and the Denisovan." Science (2014): 1250368.
It would improve the quality of the paper to add at least one comparison, even if it is a simple one using the synthetic data.

ANSWER 13.
>> Such a direct comparison would be appealing but would come with a series of issues, due to the intrinsic approaches previously developed, eg by Gokhman et al. 2014, or by some of us Pedersen et al. 2014 and Hanghøj et al. 2016. The main reason is because the latter approaches compute, within a given genomic window, the raw fraction of post-mortem DNA damage events normalized by the sequencing depth (this statistics is often referred to as Ms for methylation scores). While Ms values provide genuine DNA methylation measurements for a given individual individual, they are not directly comparable between individuals. This is so because different samples were affected by different post-mortem DNA deamination processes and are generally sequenced to various depths. It follows that comparing Ms values to f, the fraction provided by DamMet of CpG methylated within a genomic window, is impossible. Comparisons are only possible when sample-dependent rescaling procedures are implemented. This is not bullet-proof (as introduces its own series of potential bias) and generally requires external data, preferably WGBS data, from the exact same tissue as the ancient DNA was extracted from. To avoid that such bias could affect our validation procedure to assess the performance of DamMet, we have decided to compare DamMet to methylation data generated from fresh tissues (i.e. WGBS). This also provides a direct illustration that DamMet provides measures that are directly

comparable to the available data from fresh tissues.

However, we have added a table showing pearson correlations between the MLE of f from DamMet and Ms computed by epiPALEOMIX in the same genomic regions as shown in figure 3 for the two ancient specimens and added the following to the results section of the manuscript:

“Finally, we investigated whether DamMet retrieved regional methylation values on par with the Ms count statistic implemented in epiPALEOMIX \citep{hanghoj_fast_2016}. As Ms is not scaled and thus not directly comparable in terms of absolute values, we instead investigated the correlation of Ms and f in various genomic regions (Table \ref{tab:f_vs_ms}). We find that for both Ust-Ishim and Vi33, the two statistics are positively correlated (p-values $<1e^{-16}$). In line with our expectation, we find much lower positive correlation coefficients for Vi33 than for Ust-Ishim as epiPALEOMIX assumes that all observed CpG$\rightarrow$TpG conversions, including sequencing errors, true variants, and deamination of unmethylated cytosine residues, reflect true signals of methylation.”

POINT 14.
Q14.) I had problems in the installation. The problem was from the external package: htslib. Installing the following libs fixed the problem:
sudo apt-get install -y libbz2-1.0 libbz2-dev libbz2-ocaml libbz2-ocaml-dev liblzma-dev
It would be nice to include something similar for readers facing the same problem, probably at the Supplementary Material 1.4.

ANSWER 14.
>> We thank the reviewer for his/her suggestion. We have added this indication to the README on gitlab https://gitlab.com/KHanghoj/DamMet/blob/master/README.md#troubleshooting

POINT 15.
Q15.) It would be nice to add, in the software main help, a description of its purpose (while visualizing the arguments: "./DamMet").

ANSWER 15.
>> We have added the following text to the main help:
“DamMet is a software aimed to estimate methylation maps using HTS sequencing data underlying ancient samples. The implemented model follows a two-steps procedure. The first step obtains a Maximum Likelihood Estimate (MLE) of position-specific deamination rates at both methylated and unmethylated cytosine residues. The second step makes use of these estimates to recover a MLE of local methylation levels in a user-defined window size.”

POINT 16.
Q15.) How does the tool handle "N" symbols? In references? In reads?

ANSWER 16.
>> The software ignores all sites where an “N” is either present in the reference or in the reads. This is now explicitly stated in the ‘implementation details’ in our revised manuscript:
“Moreover, DamMet ignores 'N' nucleotides in the reference genome and sequencing data.”

POINT 17.
Q16.) it is referred in the paper that, for chromosome 1, it took 2 hours in a single CPU. This value depends on the CPU frequency, which might result in much more or less of the time for other CPUs. The CPU should be described (perhaps in Supplementary Material).

ANSWER 17.
>> See ANSWER 4.

POINT 18.
Q17.) What was the maximum peak of RAM in this case?

ANSWER 18.
>> See ANSWER 4.

POINT 19.

Q18.) A simple small logo would also be nice in the README.am (obviously, not mandatory).

ANSWER 19.
>> We are not sure what that should look like. We are definitely up for generating this so feel free to come with suggestions.

POINT 20.
Q19.) Use equations as if it was text. This means that the authors need to do punctuation. For example, adding "," or "." right after the equation.

ANSWER 20.
>> DONE

POINT 21.
Q20.) In equation 1, align (vertical align) the "sites" with the "depth".

ANSWER 21.
>> DONE

POINT 22.
Q21.) Curiosity: in Equation 10 of Supplementary Material, the factor of 0.5 that is multiplied to guarantee a uniform distribution between the two parts, can it be easily changed as a parameter to the program?

ANSWER 22.
>> As of now, this parameter is hardcoded. However, it could easily be added as a user-defined parameter to account for alleles not equally likely to be observed. In absence of experimental data providing expectations on expected allelic distributions, we decided to not open the possibility to modify this parameter yet. This could be easily added in future releases of the program. We note, however, that modifying this parameter would likely affect the necessary order of iterations to be carried out across unobserved genotypes.

POINT 23.
Q22.) In Figure 3: at the top (legend), give a space between the square and respective name to other types. Apply the same to supplementary figures: 11, 12, 13, 14 and 15.

ANSWER 23.
>> DONE

POINT 24.
Q23.) In figure 3,4,5 of Supplementary Material, I had problems to recognize which minimum of read length was. Perhaps, splitting the plots would work, or any other action as long as it can be efficiently recognized.

ANSWER 24.
>> We have increased the size of the plot and reduced the line width in order to improve readability.

POINT 25.
Q24.) line 19: "deaminatino" -> deamination;

ANSWER 25.
>> DONE

POINT 26.
Q25.) line 39: "an intuition" -> Perhaps a better word choice would clarify: "overview", "synthesis", ...

ANSWER 26.
>> We have changed it to “an overview”.

POINT 27.
Q26.) Supplementary Material -> page 2, line 2: remove extra "from".

ANSWER 27.
>> DONE

POINT 28.
Q27.) In the list of Abbreviations:
it is missing a ";" after ancient DNA.
Give a space in "PMD:post[...]" -> "PMD: post[...]".

ANSWER 28.
>> DONE

POINT 29.
Q28.) In the DamMet, several filtering steps are performed. This is highly related to the primary data analysis workflows for high-throughput aDNA:
Kircher, Martin. "Analysis of high-throughput ancient DNA sequencing data." Ancient DNA. Humana Press, 2012. 197-228.

ANSWER 29.
>> The reviewer is correct and reference to the provided literature has now been added. Implementing such filters is crucial for high-throughput DNA sequencing data underlying ancient genomes. In DamMet, three canonical filters can be modified by the user, and include Mapping quality, Base quality, and minimum read length.

POINT 30.
Q29.) Is there any control regarding the inclusion of exogenous ambiguous reads (according to the target genome)?

ANSWER 30.
>> DamMet makes use of PHRED quality scores so as to reflect the mapping quality. Ancient DNA data often consist of DNA reads of extremely limited length and include substantial levels of sequencing errors due to the presence of post-mortem DNA damage affecting the DNA base chemistry. Exogenous reads mapping perfectly to the reference genome are thus very difficult to identify, and error prone. In an effort to reduce the chance of observing exogenous reads (and thereby accounting for mis-mapping events), one can decide to rely on a mappability filter provided by users in BED-format to remove low complexity regions. This option is fully available in the current implementation of DamMet (using the option -e).

Close
