# Supplementary material for: DamMet: ancient methylome mapping accounting for errors, true variants, and post-mortem DNA damage
Source: Gigascience. 2019 Apr 20;8(4):giz025. doi: 10.1093/gigascience/giz025 (PMC6474913; doi:10.1093/gigascience/giz025)
Supplement: Response_to_Reviewer_Comments_Revision_1.pdf [file giz025_response_to_reviewer_comments_revision_1.pdf]

Author's Response To Reviewer Comments

Close

Dear editor,

We have now updated the tex file with the reference to gigaDB and added the dot missing in caption text of figure 2.

Best,
Kristian

Close
