# Supplementary material for: DamMet: ancient methylome mapping accounting for errors, true variants, and post-mortem DNA damage
Source: Gigascience. 2019 Apr 20;8(4):giz025. doi: 10.1093/gigascience/giz025 (PMC6474913; doi:10.1093/gigascience/giz025)
Supplement: Reviewer_1_Report_Original_Submission -- Alexander Peltzer — 12/2/2018 Reviewed [file giz025_reviewer_1_report_original_submission.pdf]

Reviewer Report

Title: DamMet: ancient methylome mapping accounting for errors, true variants and post-mortem DNA damage

Version: Original Submission **Date:** 12/2/2018

Reviewer name: Alexander Peltzer

Reviewer Comments to Author:

Dear authors,
thank you very much for making the method available and taking the time to perform such extensive research into this topic! I broadly separated my comments and questions in a set of questions + typos. I furthermore have some questions regarding the supplementary material, that I'd like to be included here as well.
Typos (please find these highlighted in the PDF as well, let me know if these can't be opened by you).
- p4, l 57, method' accuracy
- p5, l19 "deaminatino"
- p7, l10 missing "s"
- p7, l51 a "to" too much
- p7, l4 "analyze" => analyzes
-p7, l5 , "it can be parallelized easily per chromosome"
Questions to authors:
Q1.) I already used and applied gargammel to simulate ancient DNA sequences for some time and found it to be quite reliable. You mentioned that you had to add several adjustments to simulate methylation specific PMD patterns in your case. Can you demonstrate and/or explain a bit more in detail what makes you confident that you don't develop "egg and hen" to be compatible here? In other words: Gargammel to produce output based on a synthetic model that is then interpreted/analyzed well using DeamMet?
Q2.) Could you please provide some more details on the sample data you used? Referring to the publication is ok, but as data is e.g. uploaded in BAM/FASTQ format in some cases, I'd be happy to see the ENA/NCBI/SRA/ (whichever platform these were uploaded) in the manuscript to make the procedure more reproducible.
Q3.) Did you by any chance have a look at how much influence in methylation retrieval can be seen when data is processed to BAM level using different methods? I suppose you used the BAM files as provided by the respecitve authors, but did you have a look at this in general?
Q4.) Can you please provide runtimes for an entire sample? You only mentioned runtimes for chromosome 1 of one sample, but I miss memory/resource requirements for an entire sample, which could give users a hint on which kind of system they might need to use for an analysis in general. As you probably did an entire benchmark/runtime for internal usage (?) anyways, this shouldn't be a big mess for you to add.
Additional ideas/comments:
As this is a computational method, I was trying to compile and run the method on a small sample (LBK-

Stuttgart, just chromosome 22) and was happy to see that this resulted in interpretable results in general. As more and more researchers would probably want to use your method in the near future, I'd be happy to see the following as well:
- a stable release on GitLab (no need for Github, but a fixed release https://docs.gitlab.com/ee/workflow/releases.html)
- a bioconda recipe for your package once you have that stable release. This eases the pain of installation for many (!) users substantially, also giving you much more credit to have a nicely packaged and reproducibly usable method out there. As the requirements for your tool are already there in bioconda, you'd only have to write a small build + meta.yaml script to get this done. https://bioconda.github.io/contribute-a-recipe.html
I opened issues for these steps in your repository to let you know...
Supplementary comments:
- There are some latex issues in the supplementary material. Maybe try reducing the size of some of the formulas to fit into a single page width (or get these on a separate page in widescreen).
- In 2.1.2 You specify "All simulated BAM can be downloaded from XXXXX." - could you make these available? (also typo, "files" missing)
Overall the paper sounds pretty nice and well-designed to me, so I don't see any specific reason why this shouldn't get published once the minor remarks have been resolved. Thanks for your work!
In case you have specific questions, please let me know if this is allowed by the journal policies as well.

Level of Interest

Please indicate how interesting you found the manuscript: Choose an item.

Quality of Written English

Please indicate the quality of language in the manuscript: Choose an item.

Declaration of Competing Interests

Please complete a declaration of competing interests, considering the following questions:

- Have you in the past five years received reimbursements, fees, funding, or salary from an organisation that may in any way gain or lose financially from the publication of this manuscript, either now or in the future?
- Do you hold any stocks or shares in an organisation that may in any way gain or lose financially from the publication of this manuscript, either now or in the future?
- Do you hold or are you currently applying for any patents relating to the content of the manuscript?
- Have you received reimbursements, fees, funding, or salary from an organization that holds or has applied for patents relating to the content of the manuscript?
- Do you have any other financial competing interests?

- Do you have any non-financial competing interests in relation to this paper?

If you can answer no to all of the above, write 'I declare that I have no competing interests' below. If your reply is yes to any, please give details below.

I declare that I have no competing interests

I agree to the open peer review policy of the journal. I understand that my name will be included on my report to the authors and, if the manuscript is accepted for publication, my named report including any attachments I upload will be posted on the website along with the authors' responses. I agree for my report to be made available under an Open Access Creative Commons CC-BY license (http://creativecommons.org/licenses/by/4.0/). I understand that any comments which I do not wish to be included in my named report can be included as confidential comments to the editors, which will not be published.

I agree to the open peer review policy of the journal

To further support our reviewers, we have joined with Publons, where you can gain additional credit to further highlight your hard work (see: https://publons.com/journal/530/gigascience). On publication of this paper, your review will be automatically added to Publons, you can then choose whether or not to claim your Publons credit. I understand this statement.

Yes Choose an item.
