# Supplementary material for: DamMet: ancient methylome mapping accounting for errors, true variants, and post-mortem DNA damage
Source: Gigascience. 2019 Apr 20;8(4):giz025. doi: 10.1093/gigascience/giz025 (PMC6474913; doi:10.1093/gigascience/giz025)
Supplement: Reviewer_2_Report_Original_Submission -- Diogo Pratas — 1/25/2019 Reviewed [file giz025_reviewer_2_report_original_submission.pdf]

Reviewer Report

Title: DamMet: ancient methylome mapping accounting for errors, true variants and post-mortem DNA damage

Version: Original Submission **Date:** 1/25/2019

Reviewer name: Diogo Pratas

Reviewer Comments to Author:

Dear authors,
In my opinion, the paper addresses an important problem, is of interest, and, after careful improvement, may deserve to be published in GigaScience.
The validation uses both synthetic and real aDNA data, which, in my opinion, is a rigorous process of presenting the method.
The tool has good programming implementation and supporting material.
Generally, the article has potential.
However, before, the following concerns are raised:
The title of this paper describes a new method (and its implementation) for mapping ancient methylomes. However, I think it rather describes a model for measuring post-mortem deamination rates at methylated and unmethylated sites (and this is described in the abstract and through all article). The symmetric methylome regions can be derived by comparison with cross-samples. Therefore, in my opinion, the title might be a little confusing.
Another point is the absence of MapDamage in the article (at least the version 2), namely JÃ³nsson, HÃ¡kon, et al. "mapDamage2. 0: fast approximate Bayesian estimates of ancient DNA damage parameters." Bioinformatics 29.13 (2013): 1682-1684.
What is new regarding MapDamage2? What was used from MapDamage2? One paragraph to explain to the community the foundations would be kind, even if major changes have been applied and other sub-methods added.
In theory, the words "ancient methylomes" are referred to both symmetric and asymmetric methylation. The method seems to detect only symmetric methylations (CpG), while I do not see any reference regarding asymmetric methylation (CpNpNp), namely what occurs in plants. There are also characteristics of particular methylations, namely from invertebrates and plants. See, for example, Zemach, Assaf, et al. "Genome-wide evolutionary analysis of eukaryotic DNA methylation." Science 328.5980 (2010): 916-919. Therefore, please clarify if the method only works for the symmetric type.
In the article and Supplementary Material, I've seen that the method underestimates certain values. I've no problems with this, namely because underestimating means that when the tool reports any signal it means that it exists. Therefore, no false negatives are identified, which is better than having false positives. An example of non-overestimation is present in, for example, Pratas, Diogo, et al. "Metagenomic Composition Analysis of an Ancient Sequenced Polar Bear Jawbone from Svalbard." Genes 9.9 (2018): 445.
Still, in the underestimation, the default mapping values (bwa) have been used (only seed disabled),

which for ancient DNA, might not be appropriate. Increasing the edit distance and using relaxed thresholds might increase the mapping rate, especially in reads with length below 40. Please, see Schubert, Mikkel, et al. "Improving ancient DNA read mapping against modern reference genomes." BMC Genomics 13.1 (2012): 178.
So, I'm wondering why appropriate parameters for aDNA were not used in this article? Does this process increase the ambiguity while using DamMet? Please, be clear here.
There isn't a direct comparison with other methods, namely from Gokhman, David, et al. "Reconstructing the DNA methylation maps of the Neanderthal and the Denisovan." Science (2014): 1250368.
It would improve the quality of the paper to add at least one comparison, even if it is a simple one using the synthetic data.
Tool implementation:
* I had problems in the installation. The problem was from the external package: htslib. Installing the following libs fixed the problem:
sudo apt-get install -y libbz2-1.0 libbz2-dev libbz2-ocaml libbz2-ocaml-dev liblzma-dev
It would be nice to include something similar for readers facing the same problem, probably at the Supplementary Material 1.4.
* It would be nice to add, in the software main help, a description of its purpose (while visualizing the arguments: "./DamMet").
* How does the tool handle "N" symbols? In references? In reads?
* Computational resources:
* it is referred in the paper that, for chromosome 1, it took 2 hours in a single CPU. This value depends on the CPU frequency, which might result in much more or less of the time for other CPUs. The CPU should be described (perhaps in Supplementary Material).
* What was the maximum peak of RAM in this case?
* A simple small logo would also be nice in the README.am (obviously, not mandatory).
Mathematical style and concerns:
* Use equations as if it was text. This means that the authors need to do punctuation. For example, adding "," or "." right after the equation.
* In equation 1, align (vertical align) the "sites" with the "depth".
* A comment for further work: some notation might be excessive given the simplicity of the model.
* Curiosity: in Equation 10 of Supplementary Material, the factor of 0.5 that is multiplied to guarantee a uniform distribution between the two parts, can it be easily changed as a parameter to the program?
General style and spell:
* In Figure 3: at the top (legend), give a space between the square and respective name to other types. Apply the same to supplementary figures: 11, 12, 13, 14 and 15.
* In figure 3,4,5 of Supplementary Material, I had problems to recognize which minimum of read length was. Perhaps, splitting the plots would work, or any other action as long as it can be efficiently recognized.
* line 19: "deaminatino" -> deamination;
* line 39: "an intuition" -> Perhaps a better word choice would clarify: "overview", "synthesis", ...
* Supplementary Material -> page 2, line 2: remove extra "from".

* In the list of Abbreviations:

* it is missing a ";" after ancient DNA.

* Give a space in "PMD:post[...]" -> "PMD: post[...]".

Supplementary observations:

* In the DamMet, several filtering steps are performed. This is highly related to the primary data analysis workflows for high-throughput aDNA:

Kircher, Martin. "Analysis of high-throughput ancient DNA sequencing data." Ancient DNA. Humana Press, 2012. 197-228.

* Is there any control regarding the inclusion of exogenous ambiguous reads (according to the target genome)?

Best regards.

Level of Interest

Please indicate how interesting you found the manuscript: Choose an item.

Quality of Written English

Please indicate the quality of language in the manuscript: Choose an item.

Declaration of Competing Interests

Please complete a declaration of competing interests, considering the following questions:

- Have you in the past five years received reimbursements, fees, funding, or salary from an organisation that may in any way gain or lose financially from the publication of this manuscript, either now or in the future?
- Do you hold any stocks or shares in an organisation that may in any way gain or lose financially from the publication of this manuscript, either now or in the future?
- Do you hold or are you currently applying for any patents relating to the content of the manuscript?
- Have you received reimbursements, fees, funding, or salary from an organization that holds or has applied for patents relating to the content of the manuscript?
- Do you have any other financial competing interests?
- Do you have any non-financial competing interests in relation to this paper?

If you can answer no to all of the above, write 'I declare that I have no competing interests' below. If your reply is yes to any, please give details below.

I declare that I have no competing interests

I agree to the open peer review policy of the journal. I understand that my name will be included on my report to the authors and, if the manuscript is accepted for publication, my named report including any attachments I upload will be posted on the website along with the authors' responses. I agree for my report to be made available under an Open Access Creative Commons CC-BY license (http://creativecommons.org/licenses/by/4.0/). I understand that any comments which I do not wish to be included in my named report can be included as confidential comments to the editors, which will not be published.

I agree to the open peer review policy of the journal

To further support our reviewers, we have joined with Publons, where you can gain additional credit to further highlight your hard work (see: https://publons.com/journal/530/gigascience). On publication of this paper, your review will be automatically added to Publons, you can then choose whether or not to claim your Publons credit. I understand this statement.

Yes Choose an item.
